# A BSCB‐Penetrating Nanoplatform for Enhanced Luteolin Delivery in PRMT2‐Targeted Epigenetic Therapy of Spinal Cord Injury

**DOI:** 10.1002/advs.77460

**Published:** 2026-09-27

**Authors:** Yixuan Wang, Bo Jin, Shipian Li, Qianxi Ouyang, Yu Xiao, Jiaxin Wen, Yehua Lai, Ping Wang, Yongjun Wang, Xuejun Cui, Weian Zhang, Min Yao

**Affiliations:** ^1^ Spine Disease Institute Longhua Hospital Shanghai University of Traditional Chinese Medicine Shanghai China; ^2^ Key Laboratory of Theory and Therapy of Muscles and Bones Ministry of Education Shanghai University of Traditional Chinese Medicine Shanghai China; ^3^ Shanghai Key Laboratory of Functional Materials Chemistry East China University of Science and Technology Shanghai China; ^4^ State Key Laboratory of Discovery and Utilization of Functional Components in Traditional Chinese Medicine Institute of Interdisciplinary Integrative Medicine Research Shanghai University of Traditional Chinese Medicine Shanghai China

**Keywords:** blood‐spinal cord barrier, luteolin, PRMT2, spinal cord injury

## Abstract

Spinal cord injury (SCI) is a devastating disorder of the central nervous system (CNS) leading to irreversible neurological deficits. Effective pharmacotherapy remains elusive, due to the restrictive blood‐spinal cord barrier (BSCB), underscoring the urgent need for targeted and efficient drug delivery strategies. Luteolin (LUT), a natural flavonoid with potent anti‐neuroinflammatory properties, presents a promising therapeutic option; however, it is compromised by poor solubility, low bioavailability, and limited BSCB penetration. Drawing inspiration from the traditional Chinese medicine concept of the “orifice‐opening” effect of borneol (BO), we developed BO‐modified mesoporous silica nanoparticles (MSN‐BO@LUT) for targeted LUT delivery to the spinal cord. MSN‐BO@LUT significantly enhanced LUT delivery to the spinal cord, achieving a 286.36‐fold increase over LUT, and maintained a concentration of 802.17 ng/mL at 24 h. MSN‐BO@LUT demonstrated robust anti‐neuroinflammatory and neuroprotective effects in SCI mice, reducing microglial infiltration, promoting functional recovery, and showing favorable efficacy and safety compared with the positive control methylprednisolone. Mechanistically, released LUT targets protein arginine N‐methyltransferase, reduces toll‐like receptor 4 methylation, and inhibits nuclear factor kappa B‐mediated pro‐inflammatory signaling, thereby suppressing microglial M1 polarization. These findings suggest that MSN‐BO@LUT not only provides a developed, safe, effective nanotherapeutic strategy for SCI treatment but also serves as a translatable template for developing precision nanomedicines targeting CNS disorders.

## Graphical abstract

In this work, MSN‐BO@LUT penetrates the blood‐spinal cord barrier for luteolin delivery in spinal cord injury. The nanodrug suppresses neuroinflammation and promotes functional recovery of the spinal cord. Mechanistically, luteolin targets protein arginine N‐methyltransferase (PRMT2) to decrease toll‐like receptor 4 (TLR4) methylation, thereby inhibiting nuclear factor kappa B (NF‐κB)‐mediated pro‐inflammatory signaling and microglial M1 polarization. This identifies a PRMT2/TLR4/NF‐κB immunomodulatory axis for CNS disorders.

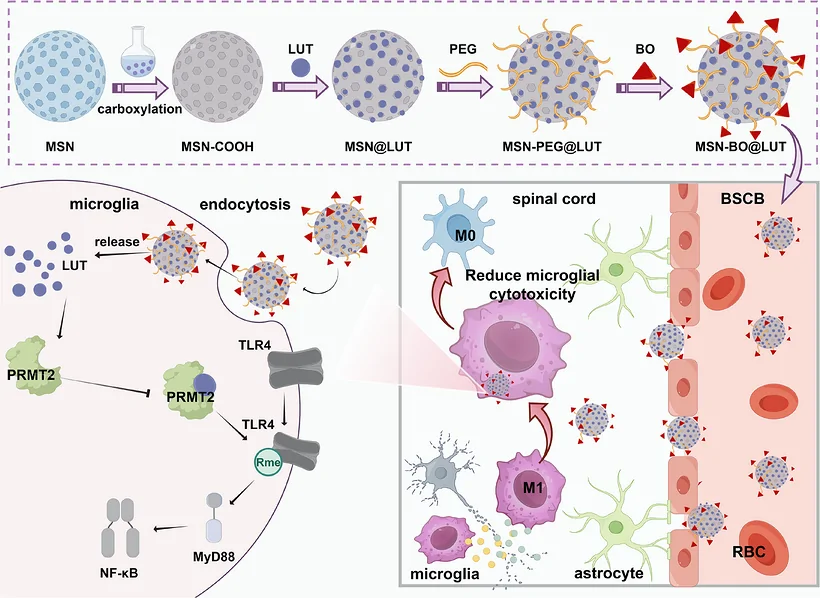

## 1 Introduction

Spinal cord injury (SCI) constitutes a traumatic disease of the central nervous system (CNS) and is characterized by the loss of motor and sensory functions below the level of injury. This loss leads to a high disability rate and serious complications [1]. The management of SCI is hindered by a critical shortage of effective treatment options, intertwined with the severe disability typical of SCI, which in turn generates a disproportionately high socioeconomic burden [2, 3, 4]. The “inflammatory storm,” which occurs following the injury, represents the central pathological characteristic of SCI. Activated microglial cells and the infiltration of peripheral immune cells (such as macrophages and neutrophils) together promote the release of pro‐inflammatory cytokines. This, in turn, exacerbates neuronal apoptosis, leading to a sustained inflammatory microenvironment within the spinal cord [5, 6, 7, 8]. Current clinical treatment strategies predominantly involve methylprednisolone (MP) pulse therapy, which can temporarily suppress the inflammatory response. However, its prolonged use can lead to significant adverse effects, including an elevated risk of infections and metabolic disturbances [7]. The development of alternative therapies and new drugs encounters challenges stemming from a key physiological structure, the blood‐spinal cord barrier (BSCB), which functions as a highly selective filter and presents significant obstacles for clinical translation [9, 10]. Consequently, there is an urgent need to develop new therapeutic strategies or more effective BSCB‐penetrating drugs tailored for SCI treatment.

Natural bioactive compounds derived from traditional Chinese medicine (TCM) offer a promising avenue for the development of novel treatments for neuroinflammation owing to their safety and natural origins [11, 12]. *Artemisia anomala*, also known as Liu‐Ji‐Nu in TCM, is extensively utilized in orthopedics to treat acute injuries. It embodies TCM theoretical principles such as promoting blood circulation, resolving stasis, and alleviating pain. However, the pharmacological basis and mechanism of action of *Artemisia anomala* are not well understood, which hampers its application in modern medicine [13, 14, 15]. Luteolin (LUT), a bioactive flavonoid isolated from *Artemisia anomala*, has gained widespread usage as a medicinal ingredient and has been effective in mitigating neuroinflammation and oxidative stress in multiple studies [16, 17]. Evidence currently demonstrates that LUT inhibits microglial polarization and reduces the release of inflammatory factors such as IL‐1β by modulating NF‐κB signaling pathways in vitro. However, the pharmacological targets of LUT remain elusive, indicating a significant gap in our knowledge [18, 19]. Moreover, the development of LUT for SCI treatment faces a primary challenge: its limited delivery to spinal cord tissue due to the BSCB [20, 21].

The traditional method of local drug administration to the spinal cord is intrathecal injection. This technique bypasses the blood‐brain barrier (BBB) and delivers the drug directly to the target site. However, it is invasive, technically challenging, and carries risks of infection and nerve damage [22, 23]. Additionally, controlling drug diffusion within the cerebrospinal fluid is challenging and often results in poor targeting [24, 25]. Nanodrug delivery systems offer potential for treating SCI. However, issues such as inadequate targeting and transient anti‐inflammatory effects pose significant challenges, underscoring the need for precise and durable delivery strategies [26, 27]. The representative drug of opening orifices in the TCM concept, borneol (BO), has aroused our attention. BO, a natural small molecule with a unique bicyclic monoterpene structure, demonstrates exceptional BBB penetration, potentially enhancing the accumulation of brain‐targeted nanoparticles by 8–12 times [28, 29, 30]. Given the structural and functional similarities between the BBB and BSCB [31], we postulated that BO modification could improve the biodistribution of nanodrugs. This approach might provide a feasible method for precise, sustained, and minimally invasive treatment in the spinal cord area.

**FIGURE 1:**
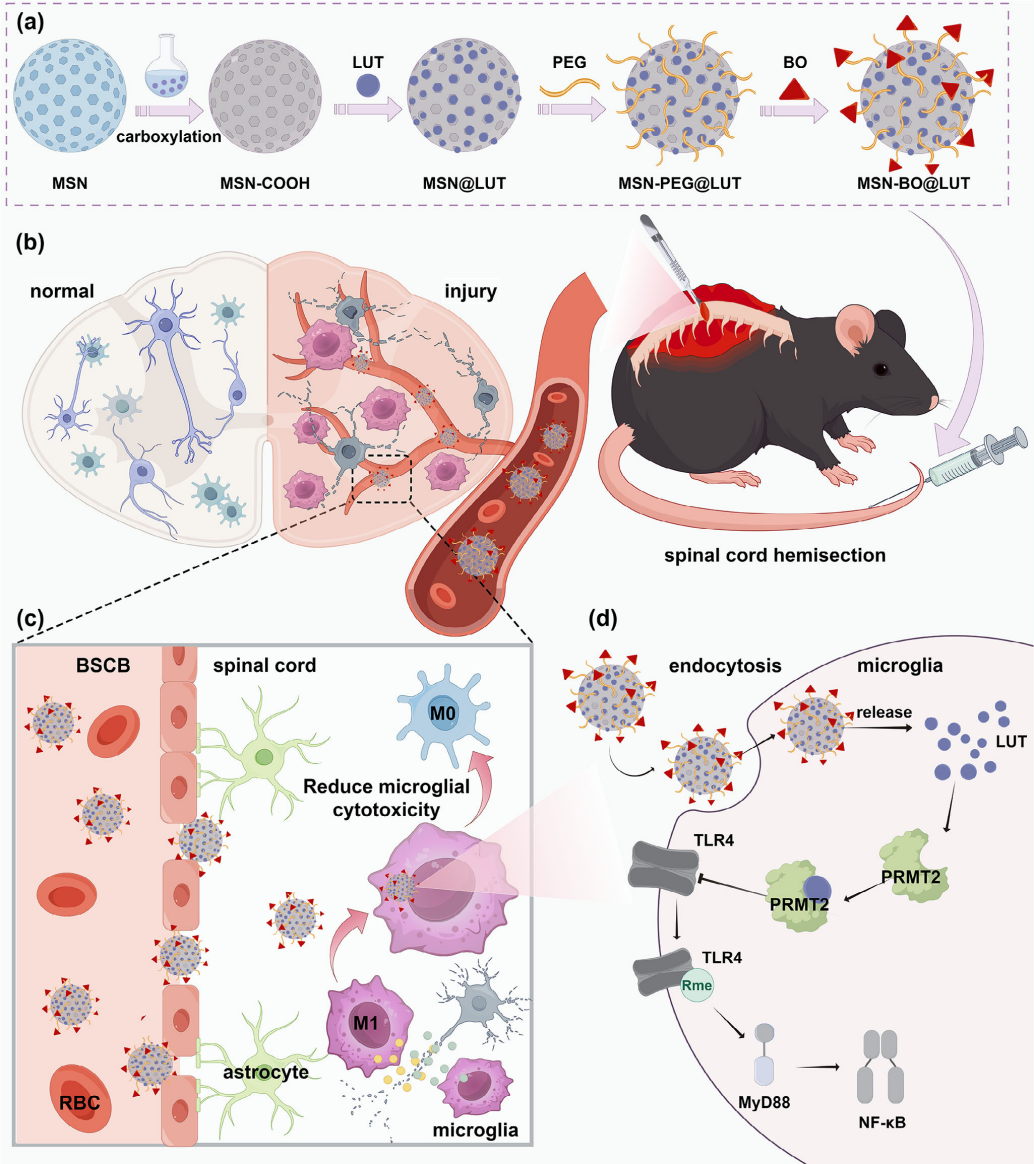
Schematic representation of the MSN‐BO@LUT nanodrug designed for BSCB penetration and amelioration of spinal cord inflammation. (a) Synthesis of MSN‐BO@LUT. (b) Establishment of a mouse spinal cord hemisection model and tail vein injection of MSN‐BO@LUT. (c) MSN‐BO@LUT penetrates the BSCB and promotes polarization of microglia from M1 to M0 phenotype under its therapeutic effect. d Internalization of MSN‐BO@LUT by microglia.

In this study, we developed a novel nanoplatform, MSN‐BO@LUT, that integrates barrier penetration, drug loading, and anti‐inflammatory properties. Mesoporous silica nanoparticles (MSNs) served as the carrier matrix, with BO covalently grafted onto the surface and LUT loaded into the mesoporous channels. This configuration enables synergistic interaction between BO and LUT (Figure 1). This design capitalizes on the attributes of each component: MSNs provide a high surface area and ordered pores for the efficient and prolonged release of LUT; BO facilitates enhanced penetration across the BSCB; and LUT acts to modulate neuroinflammatory responses at the injury site as an anti‐inflammatory agent. The MSN‐BO@LUT showed improved BSCB penetration and prolonged local anti‐inflammatory activity, effectively harnessing LUT's therapeutic potential. To assess the performance and therapeutic efficacy of MSN‐BO@LUT, we conducted a comprehensive evaluation of its physicochemical properties and drug release behavior. Subsequently, we examined cellular uptake, barrier penetration, and microglial polarization in vitro. We further investigated the targeted distribution, anti‐inflammatory effects, and neurofunctional recovery in vivo. Lastly, we elucidated LUT's target sites and the underlying mechanisms using a protein chip and multilevel transcriptomics. Collectively, this research presents a precise and promising strategy for SCI treatment, addressing crucial unmet clinical needs.

## 2 Results

### 2.1 Preparation and Characterization of MSN‐BO@LUT

MSNs were selected for drug delivery due to their tunable mesopores and large specific surface area, which are critical features for achieving high‐capacity loading and controlled release of LUT in this work [32]. MSNs were prepared via a sol‐gel process involving the condensation reaction of tetraethyl orthosilicate (TEOS) as the precursor, cetyltrimethylammonium bromide (CTAB) as the structure‐directing agent, and triethylamine (TEA) as the alkaline catalyst. Carboxyl‐modified MSNs were loaded with LUT and subsequently grafted with hydrophilic polyethylene glycol (PEG) chains. Thereafter, carboxylated BO was conjugated to the amino termini of PEG to fabricate the nanoplatform designated as MSN‐BO@LUT. This nanoplatform integrates drug‐loading (MSN), barrier‐penetration (BO), and anti‐inflammatory (LUT) modules, enabling synergistic SCI therapy (Figure 1a).

**FIGURE 2:**
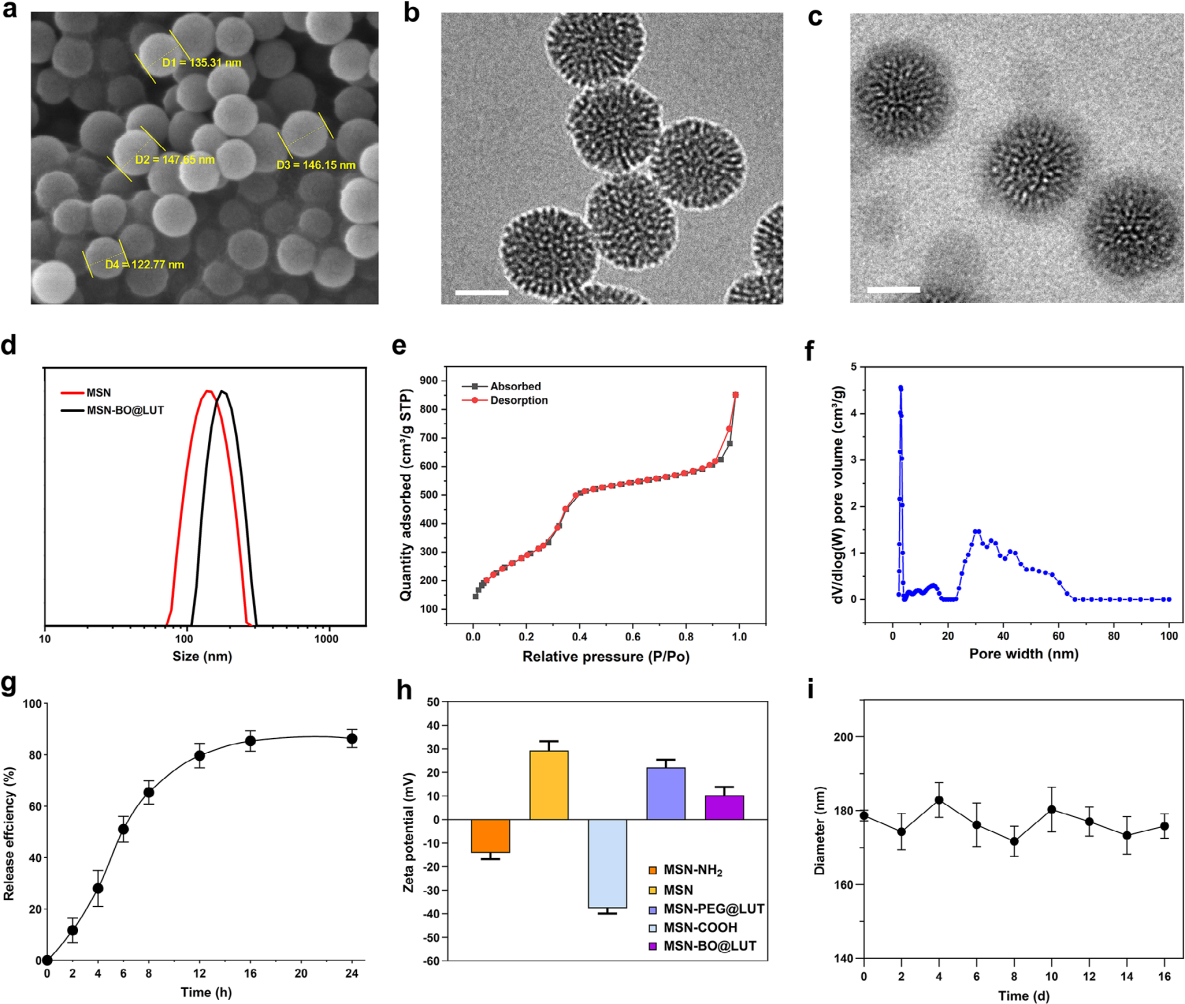
Preparation and characterization of MSN‐BO@LUT. (a) Representation SEM image of MSN. (b,c) Representation TEM images of MSN@LUT (b) and MSN‐BO@LUT (c). Scale bars = 50 nm. (d) DLS of MSN and MSN‐BO@LUT. (e) Nitrogen adsorption/desorption isotherms of MSN. (f) Pore diameter of MSN. (g) The releasing efficiencies of MSN‐BO@LUT in PBS (pH 7.4), *n* = 3 independent experiments. (h) Zeta potential of MSN, MSN‐NH_2_, MSN‐COOH, MSN‐PEG@LUT, and MSN‐BO@LUT, *n* = 3 replicates for each group. (i) Particle stability of MSN, *n* = 3 replicates for each group. Data in (g–i) are expressed as mean ± standard deviation (*SD*).

Scanning electron microscopy (SEM) and transmission electron microscopy (TEM) images showed that the MSNs exhibited a spherical structure with a diameter of approximately 140 nm, and their mesoporous structures were clearly distinguishable (Figure 2a, Figure S1). TEM images of MSN@LUT revealed that the mesopores of MSNs were filled with LUT. BO modification induced a notable increase in the particle size of MSN‐BO@LUT (Figure 2b, c). UV–vis absorption spectroscopy of the MSN‐BO@LUT complex displayed characteristic peaks of LUT, confirming successful drug loading within the mesoporous carriers (Figure S2). Fourier transform infrared spectroscopy (FTIR) analysis verified the presence of the silica matrix (Si‐O‐Si vibrations: asym. at 1079 cm^−1^, sym. at 800 cm^−1^) and successful surface functionalization (N‐H bending at 1642 cm^−1^, C = O stretching at 1640/1733 cm^−1^ corresponding to carboxyl groups/BO esters, and C‐H stretching at 2890 cm^−1^ attributed to PEG). Dynamic light scattering (DLS) measurements showed that the hydrodynamic diameter increased from 120 to 140 nm after PEG grafting, providing solid evidence of successful PEGylation and BO conjugation, and further verifying the formation of the target MSN‐BO@LUT complex (Figure 2d, Figure S3). The N_2_ adsorption‐desorption isotherms of MSNs conformed to the Type IV isotherm with a hysteresis loop. Capillary condensation occurred at relative pressures of 0.3–0.4, which confirmed the mesoporous nature of MSNs, with the pore size distribution centered at 40 nm (Figure 2e, f). This hierarchical structure facilitated high drug loading and sustained release. The maximum release rate reached 85% at 16 h, and the loading capacity was as high as 35.4%, which benefited from the large pore size and surface area of MSNs (Figure 2g). Successful surface engineering was further validated by zeta potential variations. Moreover, the stable colloidal performance over 7 days demonstrated that surface functionalization effectively endowed MSN‐BO@LUT nanoparticles with electrostatic stabilization (Figure 2h, i). Collectively, these results confirmed the successful surface functionalization of MSNs. The MSN‐BO@LUT was fabricated as designed and possessed favorable sustained‐release properties and colloidal stability, indicating its great potential for further in vivo validation.

### 2.2 LUT Ameliorated Neuroinflammation via Reduced M1 Microglia Polarization

**FIGURE 3:**
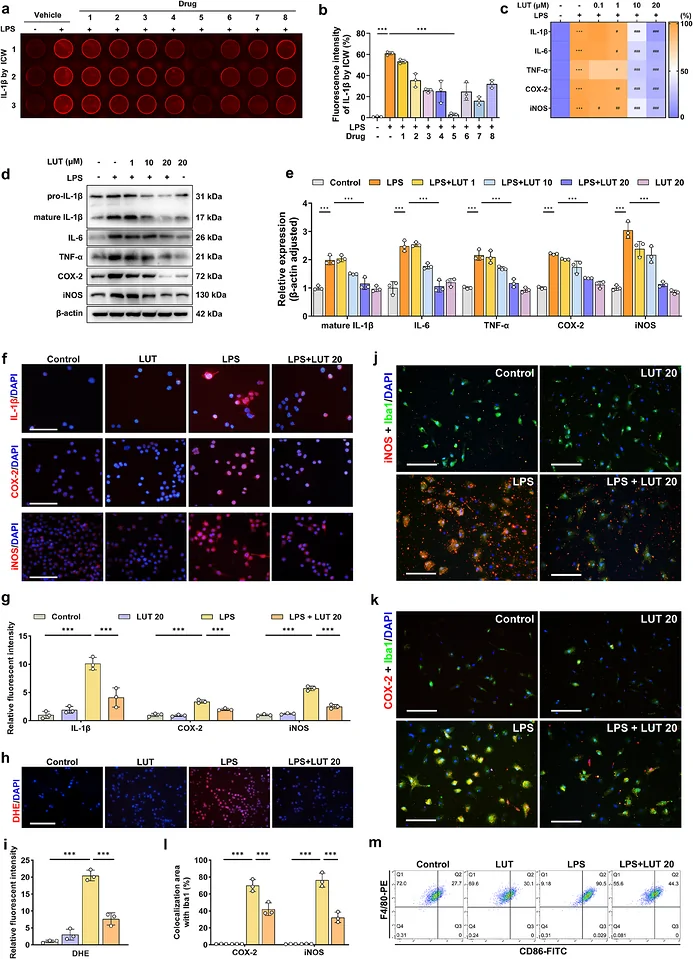
LUT ameliorated neuroinflammation via reduced M1 microglia polarization. (a, b) Fluorescence images and quantitative analysis of ICW assay for IL‐1β expression in BV2 cells treated with LPS (1 µg/mL) and active ingredients (20 µm) of *Artemisia anomala S. Moore*: isofraxidin (1), quercetin‐7‐O‐β‐D‐glucopyranoside (2), apigenin‐7‐O‐β‐D‐glucopyranoside (3), 7‐methoxycoumarin (4), LUT (5), quercetin (6), kaempferol (7), and eupatorin (8), *n* = 3 biologically independent samples, ^***^ *p* < 0.001. (c) mRNA expression levels of pro‐inflammatory factors in LPS‐activated BV2 cells treated with varying concentrations of LUT, *n* = 3 biologically independent samples. ^***^ *p* <0.001 vs. control; ^#^ *p* < 0.05, ^##^ *p* <0.01, ^###^ *p* < 0.001 vs. LPS. (d,e) Western blot and quantitative analysis of IL‐1β, IL‐6, TNF‐α, COX‐2 and iNOS expression in LPS‐activated BV2 cells treated with different concentrations of LUT, *n* = 3 biologically independent samples, ^***^ *p* < 0.001. (f,g) Representative immunofluorescence images and quantitative analysis of IL‐1β, COX‐2 and iNOS proteins in LPS‐activated BV2 cells treated with 20 µm LUT, *n* = 3 biologically independent samples, ^***^ *p* < 0.001. Scale bars = 100 µm. (h,i) Representative DHE staining images and quantitative analysis in BV2 cells, *n* = 3 biologically independent samples, ^***^ *p* < 0.001. Scale bars = 200 µm. (j–l) Representative immunofluorescence images and quantitative analysis of iNOS and COX‐2 expression co‐localized with Iba1 in primary microglia. Scale bars = 50 µm. (m) Flow cytometry analysis of BV2 cells labeled with CD86 and F4/80. Data in (b, c, e, g, i,l) are expressed as mean ± *SD*. ANOVA with Tukey's multiple comparisons test or Dunnett's multiple comparisons test were used for the statistical comparison among multiple groups, respectively. Statistical analyses were conducted with a 95% confidence interval, and significance was defined as *p* < 0.05. The experiment was repeated independently three times with similar results in (f, h, j, k, m).

After SCI, microglia, resident immune cells in the CNS, are activated and polarized toward the pro‐inflammatory M1 phenotype, which contributes to secondary injury processes [7]. *Artemisia anomala* has been investigated as a therapeutic candidate for SCI repair due to its traditional blood‐activating and stasis‐resolving properties, as well as its well‐documented anti‐inflammatory effects [32]. Therefore, in the initial stage of this study, we employed in‐cell Western blot (ICW) to screen the major active components of *Artemisia anomala* and identified LUT as the primary candidate with potent anti‐inflammatory activity (Figure 3a, b). CCK8 assay results confirmed that LUT exerted no cytotoxicity at concentrations up to 20 µm (Figure S4). In the detection of major inflammatory factors including IL‐1β, IL‐6 and TNF‐α, LUT was verified to effectively alleviate neuroinflammation in the CNS (Figure 3c–g). Additionally, LUT significantly reduced LPS‐induced ROS release in BV2 cells (Figure 3h, i). LUT's anti‐inflammatory effect was linked to its suppression of M1 polarization, which was validated by flow cytometry quantification of CD86^+^ cells and downregulated iNOS expression in BV2 cells (Figure 3d–g, m). We observed consistent effects in primary microglia, as indicated by the reduced immunofluorescence intensity of COX‐2 and iNOS in Iba‐1^+^ cells (Figure 3j–k).

Given the prominent in vitro efficacy of LUT, its therapeutic potential was evaluated in a mouse model of SCI induced by spinal cord hemisection. The result indicated that LUT failed to significantly ameliorate motor dysfunction in SCI mice (Figure S5). Furthermore, core SCI pathologies, including spinal cavitation, gray and white matter disintegration, neuronal apoptosis, and immune cell infiltration, were not obviously improved by LUT treatment. Meanwhile, neuroinflammation and apoptotic markers remained at comparable levels between the LUT‐treated and control groups (Figures S6–S9). We speculate that this translational limitation arises from two key factors: (1) extensive first‐pass metabolism leads to a critically low systemic bioavailability (reported to be less than 15%), and (2) the partially intact BSCB integrity restricts the delivery of LUT to the injury epicenter, resulting in subtherapeutic drug concentrations in the spinal cord [33, 34, 35]. Collectively, advanced drug delivery strategies that improve BSCB penetration are required to enhance LUT bioavailability and fully exert its anti‐inflammatory efficacy in SCI treatment.

### 2.3 MSN‐BO@LUT Effectively Promotes Trans‐BSCB Transport and Increases Drug Spinal Cord Distribution and Retention

**FIGURE 4:**
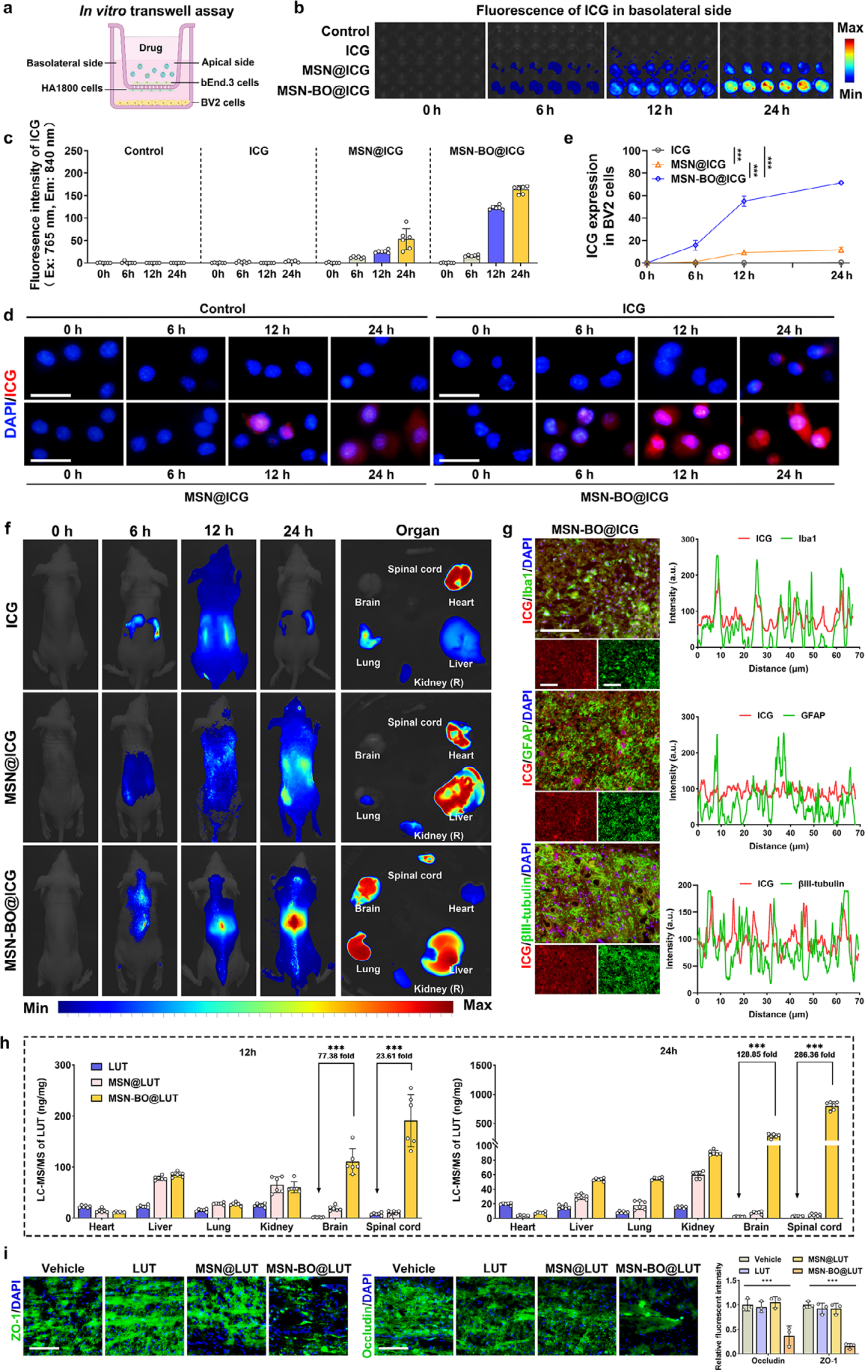
MSN‐BO@LUT effectively promotes trans‐BSCB transport and increases drug spinal cord distribution and retention. (a) Schematic of the in vitro BSCB model. (b) Fluorescence images of the medium on the basolateral side in the in vitro BSCB model after 24 h. (c) Fluorescence intensity of ICG on the basolateral side for each group, *n* = 6 biologically independent samples, excitation: 765 nm, emission: 840 nm. (d, e) Representative fluorescence images and quantitative analysis of ICG in BV2 cells on the bottom of the basolateral side, *n* = 3 biologically independent samples, ^***^ *p* < 0.001. Scale bar = 20 µm. (f) Representative in vivo fluorescence distribution images in mice captured at different time points and major organs at the injury site 24 h after tail vein injection by small animal in vivo imaging system (g) Representative images and quantitative analysis of ICG and its co‐localization with Iba1, GFAP, and βIII‐tubulin in the MSN‐BO@ICG group of mice were presented at the cross‐section of the SCI site 24 h after tail vein injection. Scale bar = 50 µm. (h) LC‐MS/MS of LUT distribution of major organs at 12 and 24 h in SCI mice treated with LUT and MSN‐BO@LUT, *n* = 6 biologically independent samples, ^***^ *p* < 0.001. (i) Representative images and quantitative analysis of tight junction proteins ZO‐1 and Occludin in the spinal cord of SCI mice, *n* = 3 biologically independent samples, ^***^ *p* < 0.001. Scale bar = 50 µm. Data in (c, e, h, i) are expressed as mean ± *SD* and analyzed using MANOVA (e), or a two‐tailed Student's *t*‐test (h, i). Statistical analyses were conducted with a 95% confidence interval, and significance was defined as *p* < 0.05. The experiment was repeated independently three times with similar results in (d) and (f–h).

For the systematical assessment of the ability of MSN‐BO@LUT to cross the BSCB, near‐infrared fluorescent indocyanine green (ICG) served as a tracer for creating MSN‐BO@ICG and control MSN@ICG. An in vitro transwell assay utilizing transwell chambers with a co‐culture of bEND.3 and HA1800 cells simulated the structure of the BSCB (Figure 4a). The in vitro transwell‐BSCB model demonstrated that both MSN‐BO@ICG and MSN@ICG could effectively penetrate the BSCB, with MSN‐BO@LUT showing significantly stronger penetrability (Figure 4b, c). The accumulation of ICG in BV2 cells on the basolateral side further confirmed that the BO modification promoted carrier penetration into the BSCB (Figure 4d, e).

Subsequent in vivo tracking in SCI mice revealed that MSN‐BO@ICG generated substantially stronger fluorescence signals at spinal lesion sites compared to controls at all observed time points (Figure 4f). Ex vivo imaging conducted 24 h post‐injection showed that while MSN@ICG primarily localized in reticuloendothelial organs such as the lungs and liver, MSN‐BO@ICG concentrated in the spinal cord, brain, and heart. This distribution pattern was also observed in uninjured mice (Figure S10). Importantly, co‐localization analysis 24 h post‐injection in the spinal cord showed that MSN‐BO@ICG nanoparticles effectively crossed the BSCB and were preferentially taken up by microglia, possibly due to their enhanced phagocytic capacity (Figure 4g and Figure S11).

To characterize the biodistribution kinetics of LUT, LC‐MS/MS quantification of LUT concentrations in multiple organs of SCI mice was performed at predetermined time points following administration of MSN‐BO@LUT, LUT alone, and MSN@LUT. As revealed by the result, MSN@LUT and MSN‐BO@LUT significantly prolonged LUT's retention time in the respective organ (Figure 4h). However, MSN@LUT alone exhibited a limited ability to cross the BSCB. Remarkably, MSN‐BO@LUT mediated explosive drug accumulation at the injury epicenter: the drug concentration in the spinal cord increased by 23.61 times at 12 h and by 286.36 times at 24 h compared to that of free LUT. This significant increase in both peak concentration and sustained exposure, which aligned with the ICG tracking data, suggested that BO modification enables the nanocarriers to efficiently target the spinal cord in vivo. MSN‐BO@LUT can effectively penetrate the BSCB and facilitate the entry of LUT into the spinal cord parenchyma in the injured area. Accordingly, drug distribution and retention time in the spinal cord can be significantly enhanced, which is of high importance for subsequent SCI treatment. The expression of tight junction proteins in primary meningeal endothelial cells and in the spinal cord of mice was assessed in vivo for exploring the BSCB modulation mechanism. MSN‐BO@LUT treatment significantly downregulated these proteins and disrupted the continuity of ZO‐1 and Occludin, indicating a transient opening of the BSCB (Figure 4i and Figure S12). In contrast, no such changes were observed in the MSN@LUT group, confirming that BO modification specifically enables the modulation of BSCB permeability. We also assessed ZO‐1 and Occludin expression 7 d after stopping MSN‐BO@LUT treatment in injured spinal cords of SCI mice. Their levels had fully recovered to baseline, confirming that MSN‐BO@LUT–induced blood‐spinal cord barrier opening is fully reversible (Figure S13).

### 2.4 MSN‐BO@LUT Exhibited Favorable Anti‐Inflammatory Effects

**FIGURE 5:**
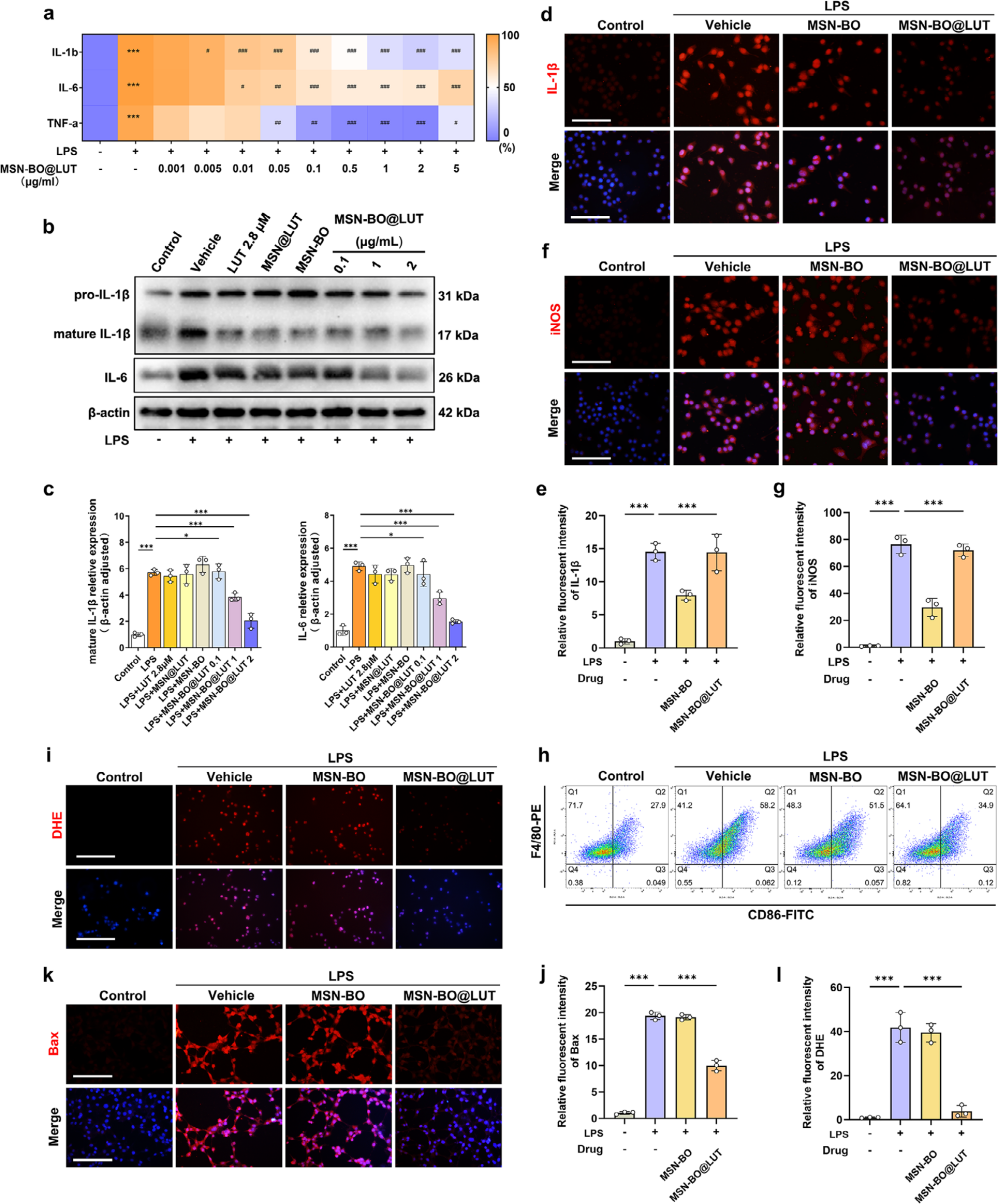
MSN‐BO@LUT exhibited favorable anti‐inflammatory effects. (a) mRNA expression of IL‐1β, IL‐6 and TNF‐α in LPS‐activated BV2 cells treated with different concentrations of MSN‐BO@LUT, *n* = 3 biologically independent samples, ^***^ *p* <0.001 vs. control, ^#^ *p* < 0.05, ^##^ *p* <0.01, ^###^ *p* < 0.001 vs. LPS. (b,c) Western blot and quantitative analysis of IL‐1β and IL‐6 in LPS‐activated BV2 cells treated with different concentrations of MSN‐BO@LUT, *n* = 3 biologically independent samples, ^*^ *p* < 0.05, ^**^ *p* < 0.01, ^***^ *p* < 0.001. (d–g) Representative immunofluorescence images and quantitative analysis of (d, e) IL‐1β and (f, g) iNOS expression in LPS‐activated BV2 cells treated with 2 µg/mL MSN‐BO@LUT and 1.2 µg/mL MSN‐BO. Scale bar = 100 µm. (h) Flow analysis of CD86 and F4/80 labeled BV2 cells. (i, j) Representative DHE staining images and quantitative analysis in BV2 cells, *n* = 3 biologically independent samples, ^***^ *p* < 0.001. Scale bars = 200 µm. (k, l) Representative fluorescence images and quantitative analysis of Bax expression in HT22 cells on the basolateral side of the in vitro transwell model, *n* = 3 biologically independent samples, ^***^ *p* < 0.001. Scale bar = 100 µm. Data in (a, c, e, g, j, i) are expressed as mean ± *SD* and analyzed using ANOVA with Tukey's multiple comparisons test or Dunnett's multiple comparisons test. Statistical analyses were conducted with a 95% confidence interval, and significance was defined as *p* < 0.05. The experiment was repeated independently three times with similar results in (d, f, i, k).

MSN‐BO@LUT showed a favorable safety profile at 10 µg/mL and effectively reduced LPS‐induced inflammation in a concentration‐dependent manner (Figure 5a, and Figure S14). Notably, MSN‐BO@LUT exhibited concentration‐dependent suppression of key pro‐inflammatory factors (IL‐6, IL‐1β) at both transcriptional and translational levels. Crucially, under matched drug‐loading conditions, MSN‐BO@LUT achieved a better anti‐inflammatory effect compared with LUT (Figure 5b–e). Furthermore, MSN‐BO@LUT significantly suppressed microglial M1 polarization, as indicated by reduced iNOS immunofluorescence and decreased CD86^+^ cells, and also inhibited LPS‐induced ROS production in BV2 cells, whereas MSN‐BO showed negligible effects (Figure 5f–j). Furthermore, an in vitro co‐culture system of BV2 and HT22 cells was established using a transwell model (Figure S15a). Following LPS stimulation of BV2 cells seeded in the upper chamber, the secreted inflammatory cytokines induced apoptosis in the underlying HT22 cells. However, treatment with MSN‐BO@LUT in the upper chamber effectively mitigated this neurotoxicity (Figure 5k, l, and Figure S15b–d). The above‐mentioned data clearly demonstrate that MSN‐BO@LUT exerts strong anti‐inflammatory effects. In this system, LUT acts as the core anti‐inflammatory pharmacophore, and BO serves as an adjuvant that enhances drug delivery without directly contributing to the observed therapeutic efficacy. Therefore, the unique structure of MSN‐BO@LUT facilitates the anti‐inflammatory activity of LUT.

### 2.5 MSN‐BO@LUT Effectively Suppresses Post‐SCI Neuroinflammation and Enhances Functional Recovery of the Spinal Cord

**FIGURE 6:**
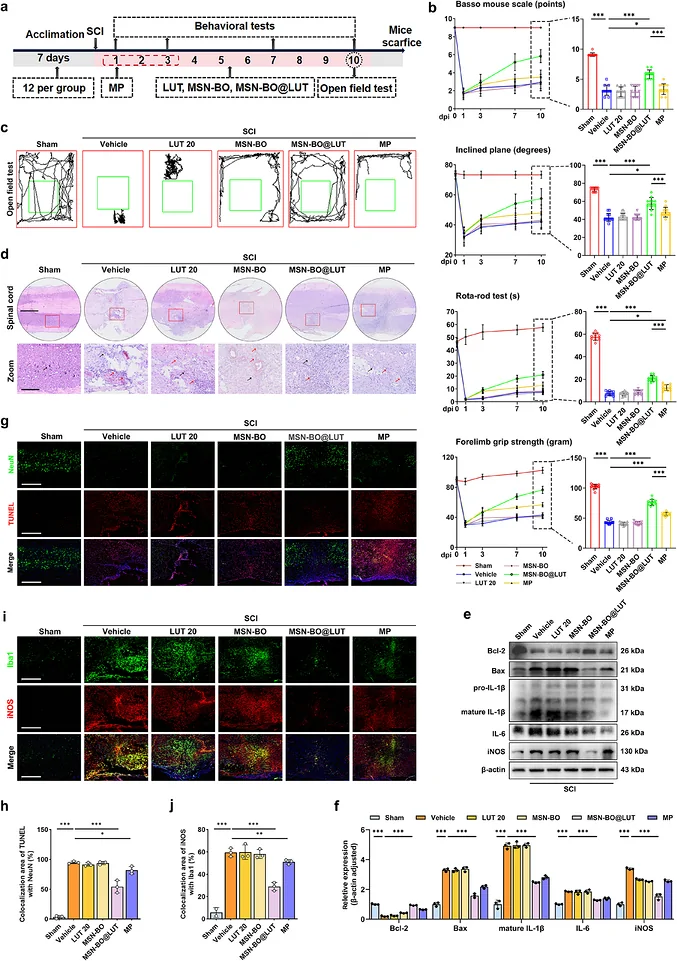
MSN‐BO@LUT effectively suppresses post‐SCI neuroinflammation and enhances functional recovery of the spinal cord. (a) Schematic diagram describing the experimental protocol. (b) BMS score, inclined plane test, rotarod test, and forelimb grip strength assessments over time, with *n* = 12 biologically independent samples. (c) Representative movement trajectory images of each mouse group in the open‐field test at 10 dpi. (d) HE staining of halved spinal cord sections from mice in each experimental group. Scale bars: 500 and 50 µm. (e, f) Western blot and quantitative analysis of Bcl‐2, Bax, IL‐1β, IL‐6, and iNOS in the spinal cord hemispheres of each group, *n* = 3 biologically independent samples. Significance markers include ^*^ *p* < 0.05, ^**^ *p* < 0.01, ^***^ *p* < 0.001. (g–j) Representative immunofluorescence images and quantitative analysis of TUNEL‐positive cells co‐labeled with NeuN (g, h) and iNOS expression labeled with Iba1 at SCI sites (i, j). Scale bar: 500 µm. Data shown in (b, f, h, j) are expressed as mean ± *SD* and were analyzed using ANOVA with either Tukey's multiple comparisons test or Dunnett's multiple comparisons test. Statistical analyses were conducted with a 95% confidence interval, and significance was set at *p* < 0.05. Each experiment was conducted independently three times, yielding similar results in (g,i).

We further explored the safety and therapeutic efficacy of MSN‐BO@LUT in SCI mice (Figure 6a). MSN‐BO@LUT treatment significantly promoted motor recovery in SCI mice in multiple measures, as verified by comprehensive behavioral assessments: higher BMS scores, increased inclined plane test angles, prolonged retention time in the rotarod test, enhanced grip strength, as well as improved movement complexity and longer travel distances in the open field test, collectively demonstrating favorable therapeutic efficacy relative compared with MP (Figure 6b, c, and Figure S16). No significant differences in body weight were observed among all groups, and no notable differences were detected in the weight of major organs in mice after 10 days of MSN‐BO@LUT treatment (Figure S17a, b). MSN‐BO@LUT was well tolerated over the 10‐day treatment period. Histopathological examination and serum biochemical analysis (ALT, AST, Cr, BUN, CREA) of major organs (heart, liver, kidney) revealed no obvious toxic manifestations (Figures S17c and S18). Furthermore, direct safety comparison between MSN‐BO@LUT and MP confirmed that neither treatment induced detectable clinical toxicity (Figure S19).

As indicated by histopathological analysis, MSN‐BO@LUT treatment markedly ameliorated neuronal loss and inflammatory cell infiltration at the injury site in SCI mice, which correlated with the observed behavioral recovery (Figure 6d). Furthermore, MSN‐BO@LUT significantly suppressed neuronal apoptosis, as evidenced by a reduction in TUNEL‐positive cells and the downregulation of cleaved caspase‐3 expression (Figure 6e–h, and Figure S20). Concurrently, the treatment attenuated neuroinflammation by reducing the levels of pro‐inflammatory cytokines and decreasing iNOS expression, as confirmed by both Western blot and immunofluorescence analyses (Figure 6e, f, h, i and Figure S21). The above‐mentioned findings collectively demonstrate that MSN‐BO@LUT can inhibit spinal neuroinflammation by suppressing microglial M1 polarization. Microglia‐driven neuroinflammation and neuronal apoptosis form a vicious cycle that exacerbates the inflammatory microenvironment in the injured spinal cord. MSN‐BO@LUT counteracts this pathological damage by modulating microglial polarization and repressing neuroinflammation, thereby ameliorating the inflammatory microenvironment and promoting functional neurological recovery after SCI.

### 2.6 MSN‐BO@LUT Releases LUT, Thereby Inhibiting Microglial M1 Polarization via Suppression of the TLR4/MyD88/NF‐κB Signaling Pathway

**FIGURE 7:**
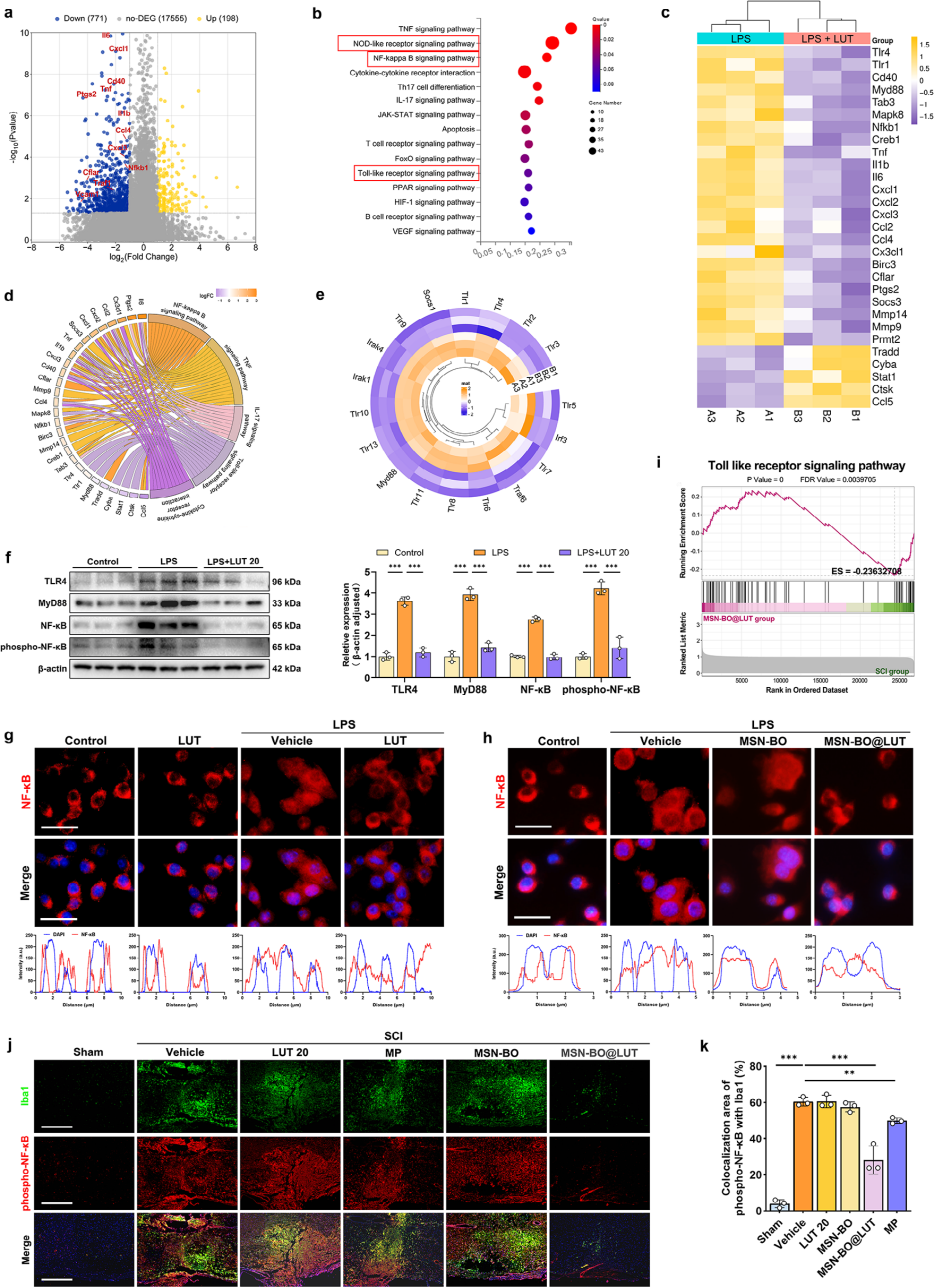
MSN‐BO@LUT releases LUT, thereby inhibiting microglial M1 polarization via suppression of the TLR4/MyD88/NF‐κB signaling pathway. (a) Volcano plot representing DEGs from RNA‐seq of LPS‐activated primary microglia treated with or without 20 µm LUT. (b) KEGG pathway enrichment analysis of DEGs. (c) Heatmap depicting DEGs associated with neuroinflammation. (d) Enriched pathway chords of DEGs and related signaling networks. (e) Heatmap focusing on DEGs related to toll‐like receptor pathways. Data in (a–e) are derived from RNA‐seq of LPS‐activated primary microglia treated with or without 20 µm LUT, *n* = 3 biologically independent samples per group. (f) Western blot and quantitative analysis of components of the TLR4/MyD88/NF‐κB signaling pathway in LPS‐activated BV2 cells treated with LUT, *n* = 3 biologically independent samples, ^***^ *p* < 0.001. (g,h) Representative immunofluorescence images and quantitative analysis of NF‐κB expression in LPS‐activated BV2 cells treated with (g) LUT and (h) MSN‐BO@LUT. Scale bar: 30 µm. (i) GSEA of significantly differentially expressed signaling pathways from bulk‐RNA sequencing of spinal cord tissue of SCI mice treated with or without MSN‐BO@LUT. (j, k) Representative immunofluorescence images (j) and quantitative analysis (k) of phosphorylated NF‐κB expression at SCI sites labeled with Iba1 for each group. Scale bar: 500 µm. Data in (f, k) are expressed as mean ± *SD* and analyzed using ANOVA with either Tukey's or Dunnett's multiple comparisons tests. Statistical analyses were conducted with a 95% confidence interval, and significance was defined as *p* < 0.05. The experiment was independently repeated three times with consistent results in (g, h, j).

To further explore the pharmacological mechanism of LUT, bulk RNA‐seq analysis of LPS‐stimulated primary microglia was performed. The results showed that LUT specifically downregulated gene clusters associated with neuroinflammation, with differentially expressed genes (DEGs) enriched in the Toll‐like receptor, NF‐κB, and NOD‐like receptor signaling pathways (Figure 7a–c, and Figure S22). Given that the NF‐κB and NOD‐like receptor signaling pathways serve as downstream cascades of the Toll‐like receptor signaling pathway, further analysis of DEGs revealed that LUT significantly inhibited the transcriptional levels of TLR4 and its adaptor protein MyD88, which attracted our research attention (Figure 7d, e). Consistent with our expectations, Western blot and immunofluorescence analyses further verified at the protein level that LUT specifically regulates the TLR4/MyD88/NF‐κB signaling axis. LUT exerts regulatory effects by not only reducing the expression of TLR4 and MyD88 but also suppressing NF‐κB phosphorylation and blocking LPS‐induced NF‐κB nuclear translocation (Figure 7f, g).

To further validate the above findings, a total of 2,252 DEGs were identified in spinal cord tissues from the MSN‐BO@LUT‐treated group and the SCI model group. These DEGs were enriched in pro‐inflammatory pathways, including the Toll‐like receptor, TNF, NF‐κB, and NOD‐like receptor signaling pathways, which was consistent with the RNA‐seq results of primary microglia treated with LUT in vitro (Figure S23). Further GSEA analysis indicated that MSN‐BO@LUT administration markedly altered the expression of gene sets related to the Toll‐like receptor signaling pathway in the injured spinal cord tissues of SCI mice (ES = ‐0.236, FDR = 0.000; Figure 7i). As a core downstream effector molecule of the Toll‐like receptor pathway and a key promoter of microglial M1 polarization, we demonstrated that MSN‐BO@LUT treatment significantly restrained LPS‐induced NF‐κB nuclear translocation and reduced the expression of phosphorylated NF‐κB in Iba1^+^ microglia within the SCI area (Figure 7h,j,k). Collectively, both in vitro and in vivo experimental results demonstrate that MSN‐BO@LUT alleviates post‐SCI neuroinflammation by releasing LUT to inhibit the TLR4/MyD88/NF‐κB signaling axis.

### 2.7 LUT Exerts its Anti‐Inflammatory Effects by Targeting PRMT2 to Inhibit TLR4 Methylation

**FIGURE 8:**
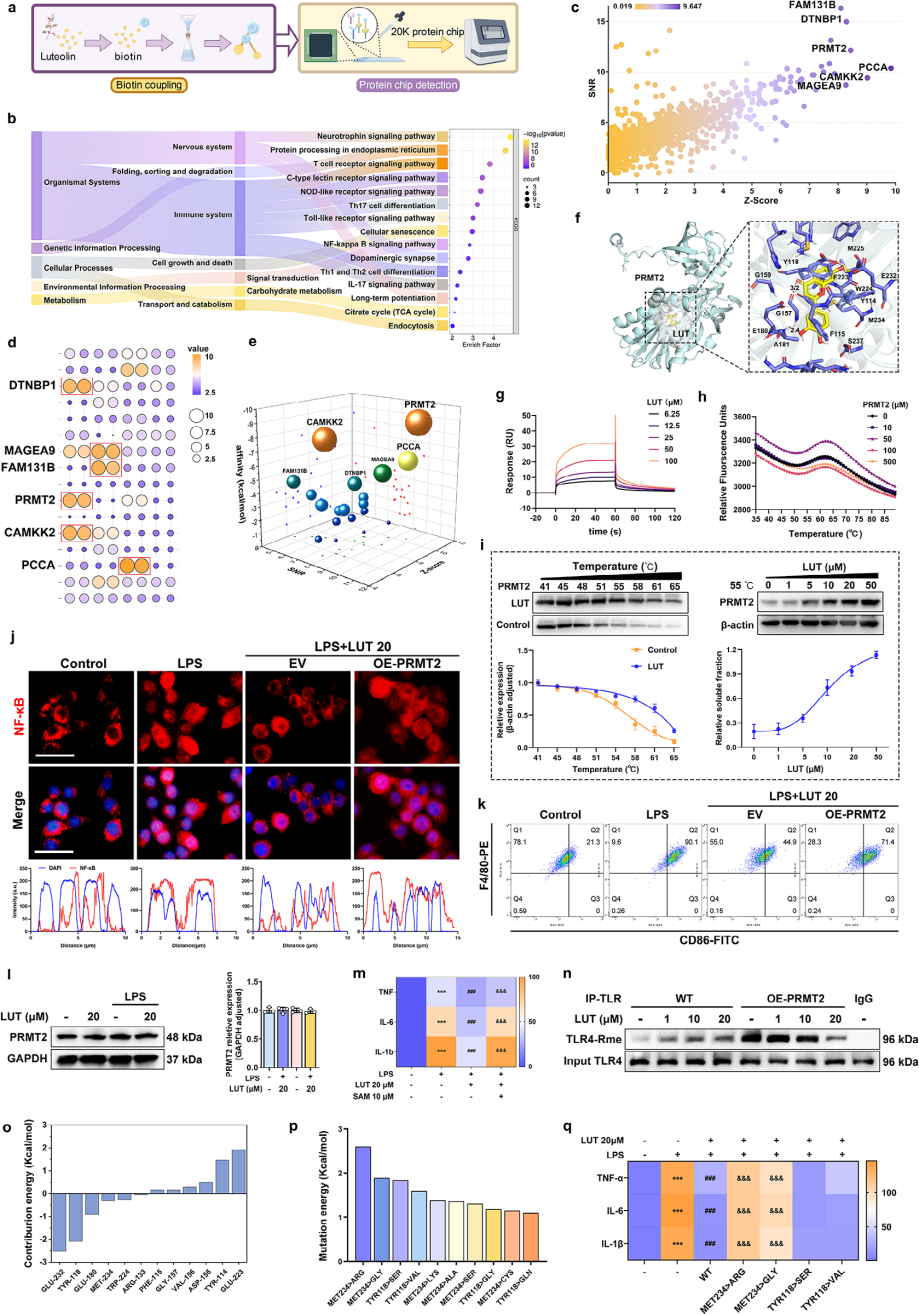
LUT exerts its anti‐inflammatory effects by targeting PRMT2 to inhibit TLR4 methylation. (a) Schematic diagram of HuProt human proteome array detection for LUT. (b) Sankey diagram of the detected sites identified via the combination of HuProt human proteome array detection and KEGG enrichment analysis following LUT treatment. (c) Scatter plot of fluorescence intensity from HuProt human proteome array detection based on Z‐score and SNR. (d) Schematic diagram of the location of the HuProt human proteome array. (e) 3D visualization integrating fluorescence intensity and molecular docking simulation results for the top 20 proteins on the array. (f) Molecular docking simulation images of LUT with the PRMT2 protein. (g) SPR results of LUT and PRMT2 proteins. (h) DSF results of LUT and PRMT2 proteins. i CETSA analysis and corresponding quantitative curves illustrating the binding interaction between LUT and PRMT2, with *n* = 3 biologically independent samples. (j) Representative immunofluorescence images and quantitative analysis of NF‐κB expression co‐labeled with DAPI in PRMT2‐overexpression or empty vector control BV2 cells. Scale bar = 30 µm. (k) Flow cytometry analysis of CD86 and F4/80 labeled BV2 cells. (l) Western blot images and quantitative analysis of PRMT2 expression in LPS‐activated BV2 cells with LUT treatment, with *n* = 3 biologically independent samples. (m) mRNA expression levels of IL‐1β, IL‐6 and TNF‐α in LPS‐stimulated BV2 cells treated with 20 µm LUT and/or 10 µm SAM, with *n* = 3 biologically independent samples. ^***^ *p* < 0.001 vs. control; ^###^ *p* < 0.001 vs. LPS; ^&&&^ *p* < 0.001 vs. LPS + LUT. (n) Pan‐methylation levels of TLR4 in BV2 cells detected by IP combined with Western blot analysis. (o) Binding energy contributions of critical amino acid residues calculated based on kinetic simulation trajectories. (p) Mutation energy of virtual saturation mutations determined via a binding stability‐based mutation strategy. (q) mRNA expression levels of IL‐1β, IL‐6, and TNF‐α in LPS‐stimulated BV2 cells treated with LUT and transfected with wild‐type or point‐mutated plasmids, with *n* = 3 biologically independent samples. ^***^ *p* < 0.001 vs. control; ^###^ *p* < 0.001 vs. LPS; ^&^ *p* < 0.05, ^&&^ *p* < 0.01, ^&&&^ *p* < 0.001 vs. LUT. Data in panels (i, l, m, q) are expressed as mean ± *SD* and analyzed using ANOVA followed by Tukey's multiple comparisons test or Dunnett's multiple comparisons test (for panels m and q). All statistical analyses were conducted at a 95% confidence interval, and statistical significance was defined as *p* < 0.05. All experiments in panels (i–n) were independently repeated three times with consistent results.

Both in vivo and in vitro studies have confirmed that LUT significantly suppresses the TLR4/NF‐κB signaling pathway. However, the specific molecular targets of LUT remain elusive. Consequently, high‐throughput screening using HuProt human proteome arrays was performed to identify potential molecular targets of LUT (Figure 8a). Biotin‐labeled LUT specifically bound to 187 proteins, with binding signal intensity more than three times the background level. Among these, immune‐inflammation‐related pathways exhibited significantly higher enrichment compared to other functional categories (KEGG enrichment P = 1.2 × 10^−7^, Figure 8b). Notably, protein microarray screening of the top 20 candidate targets, followed by molecular docking validation, identified protein arginine methyltransferase 2 (PRMT2) as the target with the strongest binding affinity for LUT (ΔG = −8.6 kcal/mol, Figure 8c–e). Furthermore, molecular docking revealed that LUT binds deep within the PRMT2 catalytic pocket, engaging 12 residues (Figure 8f). SPR kinetic fitting analysis determined the binding dissociation constant (KD) of LUT and PRMT2 to be 3.668 × 10^−5^ m, indicative of a high‐affinity small molecule‐protein interaction (Figure 8g). DSF analysis further demonstrated that LUT increased the Tm value of PRMT2 protein in a concentration‐dependent manner (0–500 µm) by 1.29 ± 0.14 °C, confirming that ligand binding induces a conformational shift toward a more stable state (Figure 8h). Additionally, CETSA analysis confirmed that LUT significantly protects PRMT2 protein from temperature‐induced denaturation within cells (Figure 8i). Collectively, these findings provided compelling evidence that LUT directly interacts with PRMT2.

Previous studies have indicated that PRMT2 and NF‐κB signaling are cross‐regulated [36], but specific mechanisms have yet to be confirmed. Consequently, we observed that overexpression of PRMT2 abolished the inhibitory effects of LUT on both NF‐κB nuclear translocation and M1 polarization, confirming PRMT2 as the critical anti‐inflammatory target of LUT (Figure 8j, k). Members of the PRMT family have been shown to regulate immune signaling pathways through arginine methylation [37]. Considering that PRMT2 possesses a catalytic domain and that reduced TLR4 promoter methylation correlates with increased TLR4 expression, we initially assessed the protein levels of PRMT2 [38, 39]. Western blot analysis revealed that neither LPS stimulation nor LUT treatment altered PRMT2 expression in BV2 cells (Figure 8l). To functionally test whether LUT inhibits PRMT2 activity, we treated LPS‐stimulated BV2 cells with ademetionine (SAM), a methyl donor and an activator of PRMT2. SAM reversed the suppression by LUT of TNF‐α, IL‐6, and IL‐1β mRNA levels (Figure 8m), confirming that LUT acts by limiting PRMT2‐dependent methylation. We then evaluated the pan‐methylation level of TLR4 using immunoprecipitation (IP) combined with Western blot analysis. Notably, in PRMT2‐overexpressing BV2 cells, LUT treatment resulted in a dose‐dependent reduction in TLR4 pan‐methylation (Figure 8n), suggesting that LUT may affect PRMT2‐mediated methyltransferase activity rather than altering PRMT2 expression. Furthermore, we explored specific methylation sites and observed that LUT treatment was associated with reduced methylation levels within the TLR4 IP complex in these overexpression cells (Figure S24). Collectively, these results suggest that LUT binds to PRMT2 and may contribute to reduced TLR4 methylation, thereby suppressing both the TLR4/NF‐κB signaling pathway and neuroinflammation.

To identify key residues for LUT‐PRMT2 binding, we combined molecular dynamics trajectory analysis with virtual mutagenesis. Binding free energy decomposition identified GLU‐232 (ΔG = −2.52 kcal/mol) and TYR‐118 (ΔG = −2.07 kcal/mol) as the top‐contributing residues, suggesting their role in stabilizing the complex (Figure 8o). Virtual saturation mutagenesis pinpointed four hotspot mutations (MET234→ARG/ALA; TYR118→SER/ALA; Figure 8p). Functional assays in BV2 microglia confirmed that substitutions at MET234 (ARG/ALA) abolish LUT's anti‐inflammatory efficacy, while TYR118 mutants retain partial activity, establishing MET234 as the critical residue for LUT‐PRMT2 binding and signaling inhibition (Figure 8q).

## 3 Discussion

SCI caused by mechanical trauma initiates primary neuronal death and provokes a vigorous neuroinflammatory response. This response creates a persistent, harmful inflammatory microenvironment that facilitates secondary degeneration through increased neuronal apoptosis and axonal loss [40, 41, 42]. In this particular inflammatory environment, without intervention, therapeutic measures aimed at promoting neuronal survival or repair prove largely ineffective and confer minimal benefit. Ultimately, chronic neuroinflammation leads to irreversible neurological dysfunction [43, 44, 45]. Therefore, mitigating the inflammatory microenvironment is crucial for treating SCI. However, the transport across the BSCB represents a critical bottleneck that must be urgently addressed in current SCI drug therapy research [46, 47, 48]. In this study, we introduce a modularly designed nanoparticle, MSN‐BO@LUT, capable of targeted intervention in SCI treatment. The BO‐functionalized MSN penetrates the BSCB efficiently and accurately, while the pore confinement effect facilitates sustained local release of LUT within the spinal cord. LUT serves as a precise effector module, specifically binding to the PRMT2 protein to inhibit TLR4 methylation. This disruption of the TLR4/MyD88/NF‐κB signaling axis reduces microglial M1 polarization and ameliorates the inflammatory microenvironment of the spinal cord. This modular engineering approach integrates “guidance, sustained release, and regulation” within a unified framework, representing a novel paradigm for the development of nanomedicines targeting CNS diseases.

Despite the strong anti‐inflammatory and neuroprotective properties of the natural flavonoid LUT, and its safety profile, its application in the CNS, particularly the spinal cord, is impeded by several delivery barriers, including low BSCB permeability, extensive first‐pass metabolism, and rapid systemic clearance [49, 50]. These barriers prevent the achievement of an effective therapeutic concentration of LUT in SCI mice, thereby limiting its anti‐neuroinflammatory efficacy, as confirmed by this study. To overcome these hurdles, this study draws inspiration from TCM theory. The Chinese herb BO and its terpene components effectively enhance the permeability of the BBB and BSCB [30, 51]. When BO is conjugated to MSN as a targeting module, unlike MSN@LUT, which provides prolonged LUT release but fails to enter the spinal cord effectively, MSN‐BO@LUT achieves notably higher spinal cord accumulation. This confirms the critical role of BO modification in enhancing BSCB penetration. Due to the strong hydrophobic interaction between the terpene moiety of BO and the lipid bilayer of BSCB endothelial cells, MSN‐BO demonstrates superior barrier penetration efficiency, overcoming the BSCB which LUT alone cannot readily cross (Figure 3c–e). As a naturally derived “pioneer,” BO shows advantages over many artificially synthesized targeting peptides, such as angiopeptin‐2, in promoting BSCB penetration [52]. More importantly, compared with other potential BSCB‐penetrating molecules, which are limited in quantity and vary in function, BO exhibits a clearly defined mechanism of action and a significant effect [53, 54]. We further validated this by demonstrating that MSN‐BO transiently modulates the continuity of tight junction proteins at the BSCB, leading to a reversible opening of the barrier. This work employs MSN as the core framework, providing the system with physical and chemical stability superior to that of lipid‐based carriers such as liposomes and exosomes. This stability makes the carrier resistant to degradation and easy to store. Furthermore, BO's natural origin significantly reduces the costs associated with large‐scale acquisition and modification compared to complex synthetic peptides or biological membrane purification processes, greatly enhancing its clinical translational potential. The mesoporous structure of MSN effectively loads LUT and ensures steady drug release at the injury site, preventing the rapid drug loss commonly seen in systems like liposomes due to initial burst release and maintaining a stable therapeutic concentration in the affected area [55]. In this system, BO acts solely as an efficient “penetration enhancer” without interfering with LUT's inherent anti‐inflammatory and neuroprotective pathways, ensuring precise therapeutic effects. It is worth noting that the MSN‐BO system uniquely integrates the stability and processability of inorganic nanocarriers (MSN) with BO's active targeting and BSCB penetration capability, comparable to biological membrane carriers like exosomes, while avoiding the costs, stability, and scalability issues associated with biological materials. This design strategy clearly separates the drug delivery function (BO‐mediated targeting and MSN‐mediated sustained release) from the core pharmacological effect (LUT), effectively avoiding the common problem of mutual interference between components in traditional co‐delivery systems. This approach provides a highly promising standardized template for the development of efficient, stable, and economical CNS (especially spinal cord) targeted delivery platforms. It should be noted that MP was selected as the positive control drug in this study, primarily based on the following two considerations: MP is currently the most widely used drug treatment for SCI in the acute stage, and has long served as the clinical reference standard for evaluating neuroprotective intervention measures. However, MP's clinical use is constrained by severe systemic side effects, including weight fluctuations, adrenal suppression, hepatotoxicity, and immunosuppression. Moreover, long‐term or high‐dose administration may exacerbate nerve damage [56, 57, 58, 59]. On the other hand, MP is a small molecule drug that can permeate the BSCB and enter the CNS parenchyma [57, 60]. Therefore, using MP as the control most directly reflects the differences in efficacy and safety between the existing clinical standards and the new targeted delivery system, providing clear clinical translational reference significance.

Our findings indicate that LUT specifically targets the PRMT2 protein, which may act as a crucial hub in regulating neuroinflammation. Although the regulatory functions of PRMT2 in tumors are well‐documented, its role in neuroinflammation is not thoroughly understood [61]. We demonstrated that LUT inhibits TLR4 methylation by directly interacting with PRMT2, thus disrupting the TLR4/MyD88/NF‐κB signaling pathway (Figure S25). To determine whether PRMT2 directly methylates TLR4, we performed mass spectrometry on TLR4‐IP, observing that several methylation sites within this complex were downregulated upon treatment with LUT. However, full‐length TLR4 peptides were not detected, likely due to the technical challenges associated with enriching transmembrane proteins [62, 63]. Consequently, it remains to be investigated whether these sites are located on TLR4 or other coprecipitated proteins. This outcome suggests that PRMT2 may dynamically regulate key molecules in this pathway through epigenetic modifications. These discoveries establish the PRMT2‐TLR4/NF‐κB axis as a central regulatory hub for neuroinflammation and broaden the therapeutic applications of PRMT2 inhibitors from oncology to neuroinflammation. Notably, LUT, as a natural compound, can potentially circumvent off‐target toxicity compared to synthetic PRMT2 inhibitors. Its capacity for multi‐pathway synergy offers a unique advantage for precisely modulating neuroinflammation. In particular, the critical role of PRMT2 in regulating microglial polarization presents a novel approach for targeted therapy in diseases such as Alzheimer's disease and stroke. This study demonstrates that LUT's neuroprotective effects are multifaceted, extending beyond anti‐inflammation. While confirming its anti‐inflammatory action via the PRMT2/TLR4/NF‐κB pathway, RNA‐seq and protein chip analyses also revealed effects on HIF‐1α, VEGF, neurotrophic factor‐related pathways, endoplasmic reticulum stress, and synaptic function (Figure 7b and Figure S23). Previous research supports that LUT activates Nrf2/ARE signaling to enhance antioxidant defense and PI3K/AKT signaling to promote cell survival and inhibit apoptosis, while it suppresses the MAPK pathway and upregulates neurotrophic factors, including BDNF, GDNF, NGF, and NT3, in the injured spinal cord [64, 65, 66]. Consequently, LUT alleviates oxidative stress, supports vascular repair, and modulates neurotrophic factors, enhancing the neural repair microenvironment. Its multi‐target profile provides a theoretical advantage over MP for the treatment of SCI. Future research should further investigate the dynamic regulatory mechanisms of PRMT2 on TLR4 methylation under diverse neuropathological conditions and develop innovative modulators of neuroinflammation based on the structural features of PRMT2. Additionally, protein chip results suggest that LUT may interact with other proteins, indicating broader therapeutic applications for LUT. This study focuses on benchmarking our LUT‐based system against free MPs, which are the clinical standard. Subsequent research comparing MSN‐BO@LUT with MSN‐BO@MP is required to provide a more stringent assessment of the LUT‐based system and to further evaluate its broader applicability as a carrier, although this comparison was not performed in the current study and thus represents a limitation and an important future direction.

## 4 Conclusion

This study developed BO‐modified mesoporous silica nanoparticles (MSN‐BO@LUT) for the targeted delivery of LUT to the spinal cord, offering an effective therapeutic approach for SCI. This innovation leverages the BSCB‐penetrating capabilities of a natural product combined with the stability and high surface area of an inorganic framework to facilitate efficient delivery and prolonged retention of LUT. LUT concentrations in the spinal cord increased by 286.36‐fold at 24 h, and sustained release was maintained at the lesion site. At the molecular level, LUT targets PRMT2, inhibits TLR4 methylation, and suppresses activation of the TLR4/MyD88/NF‐κB signaling pathway, which regulates microglial M1 polarization and reduces neuroinflammation. This work not only enhances understanding of PRMT2‐mediated regulation in CNS disorders but also establishes a generalizable paradigm that synergizes natural products with modular nanoplatforms. Moreover, it demonstrates the translational potential of integrating traditional herbal bioactives with nanotechnology, offering a promising strategy for precision therapy in SCI and other neuroinflammatory conditions and providing a framework for the modernization of TCM‐derived therapeutics.

## Author Contributions

Y.W. and B.J. contributed equally to this work. Y.W. and B.J. conceived the project, designed the experiments and analyzed the data. S.L., Q.O., Y.X., J.W., Y.L., and P.W. conducted the experiments and analyzed the data. W.Z., Y.W., X.C., and M.Y. helped interpret the results. All authors contributed to the writing of the manuscript.

## Funding

This work was supported by the National Key R&D Program of China (2024YFC3507401 for Yong‐jun Wang), National Natural Science Foundation of China (No. 82374476 for **Min Yao**, No. 82074454 for Xue‐jun Cui), National Major Science and Technology Projects of China (2025ZD1801400 for Yong‐jun Wang), Ningbo Top Medical and Health Research Program (NO. 2025021425 for Yong‐jun Wang) and Eastern Talent Plan Leading Project (Min Yao).

## Conflicts of Interest

The authors declare no conflicts of interest.

## Animal Ethics Statement

All animal experimental protocols in this work were reviewed and approved by the Animal Ethics Committee (Ethics No. LHERAW‐24024).

## Supporting information




**Supporting File**: advs77460‐sup‐0001‐SuppMat.docx.

## Data Availability

The data that support the findings of this study are available from the corresponding author upon reasonable request.
