# Supplementary material for: A BSCB‐Penetrating Nanoplatform for Enhanced Luteolin Delivery in PRMT2‐Targeted Epigenetic Therapy of Spinal Cord Injury
Source: Adv Sci (Weinh). 2026 Sep 27:e77460. Online ahead of print. doi: 10.1002/advs.77460 (PMC13616406; doi:10.1002/advs.77460)
Supplement: Supplementary file 1 [file ADVS-9999-e77460-s001.docx]

Supplementary information

A BSCB-penetrating nanoplatform for enhanced luteolin delivery in PRMT2-targeted epigenetic therapy of spinal cord injury

*Yixuan Wang^1,2,5^, Bo Jin^3,5^, Shipian Li^1,2^, Qianxi Ouyang^1^, Yu Xiao^1,2^, Jiaxin Wen^1,2^, Yehua Lai^4^, Ping Wang^4^,* *Yongjun Wang^1,2,*^, Xuejun Cui^1,2,*^, Weian Zhang^3,*^ & Min Yao^1,2,*^*

^1^Spine Disease Institute, Longhua Hospital, Shanghai University of Traditional Chinese Medicine, Shanghai, China. ^2^Key Laboratory of Theory and Therapy of Muscles and Bones, Ministry of Education, Shanghai University of Traditional Chinese Medicine, Shanghai, China. ^3^Shanghai Key Laboratory of Functional Materials Chemistry, East China University of Science and Technology, Shanghai, China. ^4^State Key Laboratory of Discovery and Utilization of Functional Components in Traditional Chinese Medicine, Institute of Interdisciplinary Integrative Medicine Research, Shanghai University of Traditional Chinese Medicine, Shanghai, China. ^5^These authors contributed equally: Yixuan Wang, Bo Jin.

**Corresponding Author:* *wangyongjun@shutcm.edu.cn; cuixj@shutcm.edu.cn;* [*wazhang@ecust.edu.cn*](mailto:wazhang@ecust.edu.cn)*; yaoming6147@shutcm.edu.cn*

**Table of Contents**

**Supplementary Methods······································································ 2**

**Supplementary Figures······································································ 16**

Supplementary Fig. 1···········································································16

Supplementary Fig. 2···········································································17

Supplementary Fig. 3···········································································18

Supplementary Fig. 4···········································································19

Supplementary Fig. 5···········································································20

Supplementary Fig. 6···········································································21

Supplementary Fig. 7···········································································23

Supplementary Fig. 8···········································································24

Supplementary Fig. 9·········································································· 25

Supplementary Fig. 10·········································································26

Supplementary Fig. 11·········································································27

Supplementary Fig. 12·········································································28

Supplementary Fig. 13·········································································29

Supplementary Fig. 14·········································································30

Supplementary Fig. 15·········································································31

Supplementary Fig. 16·········································································32

Supplementary Fig. 17·········································································33

Supplementary Fig. 18·········································································34

Supplementary Fig. 19·········································································35

Supplementary Fig. 20·········································································36

Supplementary Fig. 21·········································································37

Supplementary Fig. 22·········································································38

Supplementary Fig. 23·········································································39

Supplementary Fig. 24·········································································40

Supplementary Fig. 25·········································································41

**Supplementary** **Tables····························································****············42**

Supplementary Table 1·········································································42

Supplementary Table 2·········································································43

Supplementary Table 3·········································································44

**Supplementary Western blot images ··············****···········································45**

**Supplementary Methods**

**Cell culture**

The mouse microglia immortalized cell line BV2 cells was purchased from Procell (CL-4093) using DMEM/F12 medium (11320033, Gibco) containing 10% fetal bovine serum (FBS, 086-150, Wisent) and 1% penicillin/ streptomycin (PS, 15140122, Gibco). Cells were grown at 37°C in a 5% CO_2_ atmosphere. Brain endothelial cells were isolated from the cerebral cortex of 8-week-old male C57 mice. After cervical dislocation, brains were removed into cold D-Hanks' (ZSGB-BIO, China). Meninges and large vessels were removed, and cortical tissue was minced and digested with 0.1% collagenase II/30 U/mL DNase I (Beyotime, China) at 37°C for 1.5 h. The digest was centrifuged (1,000 rpm, 5 min), and the pellet was resuspended in 20% BSA (Gibco, USA) and centrifuged (1,000 × g, 20 min, 4°C). The bottom pellet was digested with 2.5% trypsin (Beyotime, China) for 20 min, stopped with DMEM/10% FBS (Gibco, USA), and centrifuged. The pellet was then resuspended in DMEM and layered onto a 50% Percoll (Sigma, USA) gradient. After centrifugation (1,000 × g, 10 min, 4°C), the micro vessel fraction was collected, washed, and seeded in complete DMEM (20% FBS, 1% PS, 100 μg/mL heparin, 1 ng/mL bFGF). Medium was changed after 24 h and then every other day. Cells reached 70% confluence on 7 d and were ready for further operations.

Primary microglia were isolated from the cerebral cortex and hippocampus of neonatal rats. Within 24h after birth, new born rats were euthanized on ice to collect the brains. The meninges and blood vessels were carefully removed before mincing the tissue into small pieces, digested with a digestion solution composed of DMEM/F12 medium and 0.25% trypsin (25200072, Gibcoin) a 1:1 ratio at 37°C for 10-15 min and terminated by adding DMEM/F12 medium containing 10% FBS. The cell suspension was then centrifuged (4°C, 1100 rpm, 3 min), resuspended the cell pellet in fresh DMEM/F12 complete medium (supplemented with 10% FBS, 1% PS). The suspension was filtered through a 70 µm cell strainer. Cells were seeded at a density of 50,000 cells/mL into poly-L-lysine (A600751, Sangon) pre-coated petri dishes containing DMEM/F12 complete medium. After 5 days (d) of culture, when obvious delamination was observed (microglia growing loosely on top of a confluent astrocyte layer), microglial purification was initiated. Cells were treated with a digestion solution composed of DMEM/F12 medium and 0.25% trypsin in a 2:1 ratio (37°C, 30-60 min). Purification was monitored under the microscope until the confluent astrocyte layer was fully detached. The purification was completed by replacing the medium with DMEM/F12 complete medium. Further operations were carried out 24 h later.

**In-cell Western (ICW)**

When the BV2 cells reached 70% confluency, they were treated with the active ingredients (20 μM) of *Artemisia anomala S. Moore* in the presence of LPS. The cells were fixed, permeabilized, and incubated with anti-IL-1β antibody (1:300, ab283818, Abcam) at 4°C overnight. Then, Goat Anti-Rabbit IgG H&L (1:500, ab150080, Abcam) was used for incubation at room temperature for 2 h. Finally, 96-well plates were scanned using a confocal laser microscope (TCS SP8, Leica), and the fluorescence intensity ratio of the target protein to the nuclear dye was analyzed to achieve in situ quantification of the cells. The average fluorescence intensity of protein-positive regions was analyzed using ImagePro Plus 6.0 software.

**Cell counting kit 8 (CCK8)**

The BV2 cells were incubated with 0-50 μM LUT for 12h, when the cells reached 70% confluency in the 96-well plate. Subsequently, 10 μL of CCK8 reagent (CK04, Dojindo) was added to each well and cultured for 1h. The absorbance (OD value) at a wavelength of 450 nm was measured using a Microplate Reader (Thermo Scientific, USA).

**Determination of mRNA**

*RT-qPCR*: BV2 cells were pretreated with or without 20 μM LUT and intervened with LPS. Total RNA was extracted from cells using an RNA extraction kit (B0004DP, EZBioscience) and the concentration of RNA was analyzed using a Nanodrop 2000. RNA was reverse transcribed to cDNA using a reverse transcription kit (EZB-RT2GQ, EZBioscience) and tested by the CFX96 Real-Time PCR Detection System (Bio-Rad, USA). β-actin was used as an internal reference to normalize the relative expression levels of the target genes. Mouse primer sequences were designed and listed in (**Supplementary Table 1**).

*RNA Sequencing Analysis*: After intervantion, the cells were lysed using lysis buffer (B0004DP, EZBioscience), and this detection was performed by BGI Genomics Co., Ltd (Shanghai, China). The total RNA was extracted from the samples and the concentration, purity, and integrity of RNA were assessed to ensure the qualification of the samples. The RNA was then fragmented and reverse-transcribed into cDNA. After end repair and adapter ligation, the RNA-seq library was constructed and high-throughput sequencing was performed using the DNBSEQ platform. The gene expression heatmap, Kyoto Encyclopedia of Genes and Genomes (KEGG) pathway analysis and Gene Ontology (GO) analysis were carried out using the MD5Checker tool.

**Western blot**

Total protein was extracted using RIPA buffer containing protease inhibitors. Concentrations were determined using a BCA kit (P0010, Beyotime). Proteins were separated by SDS-PAGE gels (Y1006, Yeasen) and transferred to PVDF membranes (IPVH00010, Millipore Billerica). After sealing with Rapid Closure Solution for 15 min at room temperature, the membranes were incubated with primary antibodies at 4 ℃ overnight. Specific information on primary antibodies and dilutions is provided in (**Supplementary Table 2**). After rinsing with TBST, the blot was incubated with HRP-labeled goat anti-rabbit IgG (1:5000, A0208, Beyotime)/HRP-labeled goat anti-mouse IgG (1:5000, A0216, Beyotime). Protein loading was checked for equality using HRP conjugated recombinant anti-β-actin antibody (1:3000, ZB15001-HRP-100, Servicebio). Data were checked using ChemiDoc XRS Plus Luminous Image Analyzer (BioRad, USA) with Enhanced Chemiluminescent Reagent (Thermo Fisher Scientific, USA).

**Immunofluorescence**

After cell processing, cells were fixed with 4% paraformaldehyde, permeabilized with 0.1% Triton X-100, and blocked with 5% BSA. Frozen sections were rewarmed and washed with PBS. Cells or tissues were incubated with primary antibodies at 4°C overnight. Specific information on primary antibodies and dilutions is provided in (**Supplementary Table 3**). Then, the following fluorescent secondary antibodies were added and incubated for 1h at 37°C: Donkey Anti-Rabbit IgG (1:500, ab150064, Abcam), Donkey Anti-Goat IgG (1:500, ab6885, Abcam). Cell nuclei were visualized using DAPI (C1002, Beyotime). Cell images were captured using an inverted fluorescence microscope (Ti2-E, Nikon, Japan), and tissue images were examined using a VS200 whole slide scanning system (XYLIS-XT720S, Olympus, Japan).

**Flow cytometry**

BV2 cells were collected and washed twice with PBS (1200 rpm, 3 min) after intervention, cells were counted and resuspended by stain buffer (abs9475-500mL, Absin) to adjust the final concentration of cells to 2×10^7^ cells/mL, added Fixable Viability Stain (565388, BD Pharmingen, FVS780) at room temperature for 15 min，add mouse Fc receptor blocker (abs9477-200T, Absin) at 4℃ for 5 min, add CD86 (abs1850146-100T, Absin, PE) and F4/80 (Absin,abs1850174-100T, APC) at 4℃ for 15 min. All procedures were protected from light, and detected by Attune Nxt flow cytometer (Invitrogen, USA), and data analysis was performed by FlowJo V10 software (BD, USA) for data analysis.

**Spinal cord hemisection model and group**

In this work, 150 C57BL/6 mice (6 weeks old, 15-18 g) used were provided by the Animal Experiment Center of Shanghai University of TCM and the experimental procedures were approved by the Animal Ethics Committee (Ethics No. LHERAW-24024). The SCI mouse model was produced using the spinal cord hemisection method. The experimental mice were anesthetized with 3% isoflurane by inhalation and placed in the prone position. The back is prepared and sterilized, followed by local infiltration anesthesia using 1% lidocaine, and a midline skin incision is then made on the back. The T10 vertebral plate was then bluntly separated to expose the T9-11 spinous processes and vertebral plates. A hemisection was made between the spinal cord and the posterior spinal vein at the right margin of T10 using ophthalmic iris scissors. The wound was disinfected with iodophor and sterile saline and then sutured layer by layer. The general condition of the mice was assessed daily to make sure that there were no infections or accidental injuries. The sham operation group only had a back incision without any other procedures. Except for the sham operation group, all groups underwent surgical modeling and treatment began the day after the operation.

To investigate the therapeutic effect of LUT on SCI mice, the mice were randomly divided into six groups, including sham group, vehicle group, low-dose (5 mg/kg), middle-dose (10 mg/kg) and high-dose (20 mg/kg) LUT group, and methylprednisolone (MP) group. Mice in the LUT treated group were intraperitoneally injected with LUT daily for 10 d. Mice in the MP group were intraperitoneally injected with MP at a dose of 5 mg/kg daily for 3 d. The sham and vehicle groups were intraperitoneally injected with normal saline.

In order to study the therapeutic effect of MSN-BO@LUT on SCI mice, the mice were randomly divided into 6 groups, including sham group, vehicle group, LUT group, MSN-BO group, MSN@LUT group, MSN-BO@LUT group, MP group. The remaining mice were given LUT (20 mg/kg), MSN-BO (6 mg/kg), MSN-BO@LUT (10 mg/kg) by tail vein injection. The MSN-BO@LUT group was given MSN-BO@LUT (10 mg/kg), and the rest is the same as before.

**Behavioral tests in mice**

The present study systematically assessed motor function and spontaneous activity in mice through a series of behavioral paradigms. Hindlimb motor function was evaluated using the Basso Mouse Scale (BMS, 0-9 points) by double-blinded observers during a 5 min behavioral session, with mean scores recorded. Coordination capacity was quantified via an inclined plane test, measuring the maximum tilt angle at which mice-maintained posture when positioned head-down (mean of three trials). Balance capability was determined through rotarod testing (3 cm diameter rod, initial speed 4 rpm with acceleration), recording latency to fall within 60 s (best performance out of three trials). Forelimb grip strength was measured using a digital dynamometer, capturing peak values from three attempts. Open field test was analyzed in a standardized open field arena (500×500×300 mm, thermostatically controlled at 20°C) via video tracking system, collecting 5 min movement parameters including total distance traveled, central zone dwell time, and central crossings. All behavioral assessments were measured separately by two individuals and repeated three times to ensure the reliability of the experiment.

**Histopathological staining**

Mouse spinal cord specimens were taken, fixed in 4% formaldehyde, dehydrated and embedded in frozen section embedding agent, and sectioned using a frozen sectioning machine. Mouse heart, liver and kidney tissues were taken, fixed in 4% formaldehyde, dehydrated and embedded in paraffin, sectioned using a paraffin slicer, deparaffinized with xylene and hydrated with a series of graded concentrations of alcohol. Sections were stained with hematoxylin and eosin (BL700B, Biosharp) and sealed with neutral gum. Tissue images were observed using a VS200.

**Determination of protein targets**

LUT was ligand-labeled with biotin, solubilized using DMSO, and formulated into a master mix with a concentration of 10 mM. The HuProt™ human proteome array was closed according to the standard procedure for microarray assays provided by CDI Microarray, and then the microarray was incubated with diluted LUT-biotin samples for 1 h at room temperature, 0.1% Cy5-Streptavidn Solution for 20 min, protected from light, GenePix 4000B scanner (Molecular Devices, USA), scanned the array at 635 nm, Power 100%.

Three-dimensional spatial data files of lignans were downloaded from the PubChem database (https://pubchem.ncbi.nlm.nih.gov). Crystal structures of key receptor proteins were downloaded from the AlphaFold protein structure database (https://alphafold.ebi.ac.uk/). Use AutoDockTools 1.5.6 to hydrogenate the ligand and receptor proteins, charge balance modifications, and set atom types. Molecular docking of receptor proteins and lignans small molecules was performed using AutoDock Vina 1.1.2. According to the protein-ligand complex binding energy results, CAMKK2, DTNBP1, FAM131B, PCCA, MAGEA9 and PRMT2 proteins were visualized and analyzed with LUT respectively.

**Cellular thermal shift assay (CETSA)**

*Temperature‐dependent profiling*: BV2 cells were pre-treated with 20 µM LUT or vehicle (0.1% DMSO) for 12 h. Cells were collected, and resuspended in PBS supplemented with protease inhibitor cocktail. Aliquots were transferred into thin-walled PCR tubes, heated for 5 min at 41, 45, 48, 51, 55, 58, 61 or 65°C, then cooled at room temperature for 5 min. Three freeze–thaw cycles (liquid nitrogen 30 s to 37°C, 60 s) followed to ensure complete membrane disruption. Lysates were clarified (4°C, 15000 rpm, 15 min), and supernatant protein concentrations were normalized to 1 µg/µL using the BCA assay. Protein expression was detected by Western blot, and the band intensity at each temperature was quantified by ImageJ to generate melt curves and identify the Tagg shift.

*Ligand‐dependent profiling at fixed temperature*: To evaluate LUT dose-dependent stabilization, BV2 cells were exposed to 0, 5, 10, 20, 30 or 50 µM LUT for 12 h. After harvesting and PBS resuspension as above, all samples were heated at the empirically determined optimal temperature (55°C) for 3 min, followed by freeze-thaw (3×) and centrifugation. The rest of the operations are the same as before.

**Protein interaction studies**

*Differential scanning fluorescence (DSF)*: the PRMT2 protein (TP318590, Origene) was mixed with a LUT (100 μM) in buffer solution and subsequently aliquoted into a 96-well plate. Thermal denaturation scanning was performed using a real-time fluorescence detection system (CFX96, Bio-Rad) with a linear heating rate of 0.1°C/s (25°C to 95°C). Fluorescence intensity variations were continuously monitored to generate temperature-dependent unfolding curves, from which the protein melting temperature (Tm) and cooperative binding coefficient (ΔTm) were derived through Boltzmann equation fitting.

*Surface plasmon resonance (SPR)*: LUT samples with different concentration gradients were obtained by diluting the 100 μM LUT liquor with PBS-P. The master mix was corrected using PBS-P running buffer containing 5% DMSO and solvent. The samples were injected into the system, and the change in resonance angle was measured in real time and quantitatively by the detection system of the SPR instrument to obtain affinity-related information. The reaction curves were fitted with the combined formula interaction model to determine the kinetic constants.

**Construction of nanomedicines**

MSN was prepared by sol gel method using TEOS as silicon source and CTAB as template. CTAB in MSN was extracted in acidic methanol 3-aminopropyltriethoxysilane (APTES) was used as a silane coupling agent to introduce amino groups on the surface of MSN. The amino group was further modified to carboxyl group by using succinic anhydride. LUT and MSN were stirred together to load LUT load LUT in the mesopores. NH2-PEG-NH2 was grafted onto the surface of MSN. Finally, carboxyl functionalized BO was grafted onto the other end of NH2-PEG-NH2.

**Characterization assay**

The particles were observed by transmission electron microscopy (JEM-2100F, JEOL, Japan) and scanning electron microscope (S-4800, HITACHI, Japan). The Fourier transform infrared (FTIR) spectrum of MSNs was recorded using an FTIR-350 spectrometer (IS50, Thermo Fisher Scientific, USA) by the KBr pellet method. The encapsulation efficiency was calculated by the following relationship:

$$\text{E}_{\text{p}}\text{\%=}\frac{\text{W}_{\text{L}}}{\text{W}_{\text{I}}}\text{×100}$$

E_p_ is the Encapsulation efficiency, W_L_ is the weight of drug loaded to NPs, W_I_ is the initial weight of drug.

TG and DTG curve were recorded using Differential Scanning Calorimeter (DSC 8500, PE Co., USA). The hydrodynamic size and Zeta potential of nanoparticles were measured using a nanoparticle size and Zeta potential analyzer (Litesizer DLSx01, Anton Paar, Austria). The nitrogen gas (N2) adsorption-desorption isotherm of MSN and surface area/pore size distribution analyzer under continuous adsorption conditions were measured using High Speed Automated Surface Area and Pore Size Analyzer (3Flex, Micromeritics, USA). The LUT release from MSN-BO@LUT was performed by dispersing nanocomposites in PBS solution (pH 5.7) with stirring at 37℃. The LUT@MSN-BO solutions were centrifuged at every designated time point (1, 2, 4, 6, 8, 12, 16 and 24 h), and the supernatant solution was collected.

***In vitro* BSCB transwell model**

An *in vitro* BSCB transwell model was constructed using bEND.3 endothelial cells, HA1800 pericytes, and BV2 microglial cells in a 24-well Transwell system (0.4 μm pore, Corning). First, 100 μL bEND.3 cells (2×10⁵ cells/mL) were seeded onto the Matrigel-coated apical side of the insert. Concurrently, HA1800 cells (2×10⁵ cells/mL) were seeded onto the basal (outer) surface of the inverted insert membrane to represent the perivascular space. The insert was then placed into the lower chamber containing 400 μL medium (same as upper chamber) to maintain osmotic balance; medium was changed every 2-3 d. After bEND.3 cell confluence, transepithelial electrical resistance (TEER) was measured. The BBB was deemed established (TEER > 200 Ω·cm²) before seeding 400 μL BV2 microglial cells (2×10⁵ cells/mL) to the lower chamber. MSN-BO@ICG, MSN@ICG, or ICG were then added to the upper chamber. Samples of 50 μL were collected from the lower chamber (containing BV2 cells) before incubation and at 6, 12, and 24 h after incubation for ICG fluorescence quantification (microplate reader, BioTek Instruments, Inc., USA) and distribution imaging (IVIS® Lumina III, Revvity, CLS136334, China). The fluorescence intensity of ICG in BV2 cells was observed using an inverted fluorescence microscope (OLYMPUS, IX73, Japan), and the average fluorescence intensity was quantitatively analyzed using ImageJ software.

***In vivo* imaging of mice**

MSN-BO@LUT-ICG, MSN@LUT-ICG, ICG were used as tracers to label various nanomedicines in order to track their distribution *in vivo*. Mice were injected with MSN-BO@ICG, MSN@ICG, and ICG in the tail vein 24 h after successful modeling, and fluorescence imaging was captured using the IVIS® Lumina III Small Animal In Vivo Imaging Detection System at 0, 6, 12 and 24 h after the injections, respectively.

**Detection of drug tissue distribution**

Liquid chromatography-tandem mass spectrometry (LC-MS/MS) was employed to determine the distribution of drugs in mouse tissues. Various mice tissues, including the heart, liver, lungs, kidneys, spinal cord, and brain, were collected, weighed, and rinsed with physiological saline. Using a fully automatic sample freeze grinder, 2.5 times the volume of PBS was added to prepare a homogeneous tissue homogenate. Acetonitrile was then added to fully precipitate the proteins, followed by centrifugation to obtain the supernatant. A LUT stock solution (10 μg/mL) was prepared with DMSO and subsequently diluted stepwise with an acetonitrile solution containing 0.1% formic acid to create seven working standard concentrations (3.99, 7.06, 32.9, 64.8, 296, 586, and 1070 ng/mL). For detection, an Ab Sciex 6500+ LC-MS/MS system (SCIEX, USA) was utilized. The mobile phase consisted of acetonitrile and 0.1% formic acid in water, with a flow rate set at 0.3 mL/min. The chromatographic column used was a Waters ACQUITY UPLC·BEH C18 (100 mm × 2.1 mm, 1.7 μm). The turbo ion spray source was configured with an ion spray voltage of 4500 V and a source temperature of 500°C. The curtain gas, nebulizer gas, and heater gas were maintained at 241.3 kPa, 413.7 kPa, and 413.7 kPa, respectively. The column temperature was kept at 40°C, and the analysis was conducted for 10 min.

**Drug safety test**

C57BL/6 mice were used for the test, the changes in body weight of the mice were recorded, and the damage of nanomedicine on the main organs was observed. After continuous administration for 10d, the heart, liver and kidney of mice in the sham group, vehicle group, MSN-BO group and MSN-BO@LUT group were weighed and subjected for histopathological observation.

Peripheral blood was collected from mice in each group. The levels of creatine kinase (CK), blood urea nitrogen (BUN), creatinine (Cr), and aspartate aminotransferase (AST) in the peripheral blood were detected using a kit from BECKMAN COULTER. All tests were completed by the Department of Laboratory Medicine at Longhua Hospital, Shanghai University of Traditional Chinese Medicine.

**Plasmid Construction and BV2 Cell Transfection**

The wild-type PRMT2 overexpression plasmid (pCDNA3.1(+) vector, with mouse PRMT2 cDNA, NCBI Ref-Seq: NM_011294.3 inserted) and four-point mutant plasmids (M234R, M234A, Y118S, Y118A) were constructed by Hanheng Biotech Company (Shanghai, China) through site-directed mutagenesis and verified by DNA sequencing. Experimental groups: untransfected control group, empty vector control group (pCDNA3.1(+)), wild-type PRMT2 group (WT), and PRMT2 mutant groups (M234R/M234A/Y118S/Y118A). All experiments were independently repeated three times.

BV2 cells were seeded in 6-well plates with 70% confluence, and DMEM/F12 was replaced before transfection. Each well was transfected with a complex containing 2 μg plasmid and 4 μL Lipofectamine 3000 (L3000015, Thermo Fisher Scientific), which was mixed and incubated in Opti-MEM for 15 min before being added to the cells. After 8-12 h of transfection, the medium was replaced with complete medium. Cells were collected 72 h after transfection for subsequent experiments.

**Methylation site detection**

After IP, the TLR4-bound beads were directly subjected to on-bead trypsin enzymatic digestion to release and cleave the adsorbed proteins into peptide mixtures. The resulting peptides were collected, desalted using C18 solid phase extraction (SPE) columns (Waters, MA), dried, and reconstituted in 0.1% formic acid (FA) for subsequent nano liquid chromatography-tandem mass spectrometry (nano LC–MS/MS) analysis. The LC–MS/MS system consisted of a Dionex™ UltiMate 3000 liquid chromatography system coupled online to a Q Exactive HF mass spectrometer (Thermo Fisher Scientific, USA). Briefly, the peptides were dissolved in 20 μL of 0.1% FA (v/v), and 3 μL of the solution was injected for detection. A 45 min linear gradient was established using 2%–95% (v/v) acetonitrile (ACN) in 0.1% FA. Raw MS data were analyzed and identified using pFind software (Version 3.1.5, Institute of Computing Technology, CAS) against the UniProt Mouse reviewed database. Carbamidomethylation (cysteine) and oxidation (methionine) were defined as common variable modifications for all samples. For methylation screening, lysine and arginine Methyl (+14.0268 Da) and D_Methyl (+28.0536 Da) were additionally set as targeted variable modifications.

**Immunoprecipitation**

BV2 cells were lysed in RIPA buffer supplemented with protease/phosphatase inhibitors. After pre-clearing with Protein A/G magnetic beads, 1 mg lysate was incubated overnight at 4°C with 5 μg anti-TLR4 antibody (ab22048, Abcam) or control IgG. Immune complexes were captured by Protein A/G beads, washed, and eluted in SDS loading buffer. Precipitates were analyzed by Western blot.

**Statistical methods**

For *in vivo* studies, mice were randomly assigned to experimental groups. The “n” values represent independent biological replicates for cell experiments or individual mice for *in vivo* experiments, with specific details provided in each figure legend. All data were expressed as mean ± *SD*. For fluorescence intensity quantification in tissue sections, four random fields of view per animal were analyzed, and the mean fluorescence intensity of these four fields was calculated for each mouse. Three mice per group were used, and the individual mouse means were then used for statistical comparisons between groups. Fluorescence co‑localization analysis was performed using ImageJ software, and the resulting intensity profiles were further processed and plotted with GraphPad Prism. Statistical significance was determined as follows: the unpaired two-tailed Student’s *t*-test for two-group comparisons, one-way analysis of variance (one-way ANOVA) for multi-group comparisons, with post-hoc tests where applicable, and multivariate analysis of variance (MANOVA) for analyses involving multiple correlated outcome measures, with *p* < 0.05 indicating a statistically significant difference. Exact *p* values are displayed in the respective figures. Statistical analysis was performed using SPSS 25.0 software (SPSS Inc., USA), GraphPad Prism10.1.2 (GraphPad Software, USA).

**Supplementary Figures**

**Supplementary Fig. 1 Morphological and thermal characterization of the synthesized MSNs.** **a** Transmission electron microscopy (TEM) image of the MSNs, revealing their spherical morphology and well-ordered mesoporous structure. The uniform channels are clearly visible, confirming the successful formation of a high-quality mesoporous architecture. **b** Thermogravimetric (TG) and derivative thermogravimetric (DTG) analyses. The significant mass loss below 100 °C is attributed to the removal of physisorbed water and the mass loss around 300 °C is attributed to the thermal decomposition of PEG and LUT. The high residual mass of 73.63% indicates the inorganic silica framework with excellent thermal stability.

**Supplementary Fig. 2 UV-Vis absorption spectroscopy analysis of MSN, LUT and MSN-BO @LUT complex.** The characteristic absorption peaks of LUT are clearly observed in the MSN-BO@LUT spectrum, confirming the successful loading of the drug into the mesoporous carriers.

**Supplementary Fig. 3 FTIR spectroscopy analysis of the step-wise surface modification and drug loading process.** The pristine MSN shows a characteristic Si-O-Si asymmetric stretching vibration at 1079 cm⁻¹. The successful amino-functionalization is indicated by the emergence of N-H stretching bands at 1642 cm⁻¹. The subsequent conversion to carboxyl groups is confirmed by the appearance of the strong C=O stretching vibration at 1640 cm⁻¹. The spectra of the final drug-loaded products demonstrate the presence of both the carrier and the drug, verifying successful loading.

**Supplementary Fig. 4 Cytotoxicity assessment of LUT on BV2 cells.** Cell viability of BV2 cells treated with different concentrations of LUT for 24 h was detected by CCK8 assay. The results indicated that LUT did not show obvious cytotoxicity at concentrations lower than 50 μM, demonstrating its safety within this concentration range for subsequent cell experiments. The data are presented as the mean ± *SD*. *n* = 6, ^*^*p* <0.05 vs 0 μM.

**Supplementary Fig. 5 Behavioral assessment of LUT administered via intraperitoneal injection in a mouse model of SCI. a** Schematic of the experimental design for SCI model and LUT treatment. **b** Behavioral tests including basso mouse scale, inclined plane test, rota-rod test and forelimb grip strength assay were performed at days 0, 1, 3, 7 and 10 post-surgery. No statistically significant improvements were observed in the LUT-treated group compared to the vehicle group across all tests and time points. **c** Representative trajectory maps from the open field test for each group. **d** Quantified parameters from the open field test, inclined distance in zone, fast time in zone, mean speed in zone and resting time in zone. The LUT-treated group did not show significant differences in these anxiety- and exploration-related metrics compared to the Vehicle group. The data are presented as the mean ± *SD*. *n* = 12, ^***^*p* <0.001 vs Sham group.

**Supplementary Fig. 6 Western blot analysis of apoptosis-related and inflammation-related proteins in the spinal cord tissue of SCI mice treated with LUT via intraperitoneal injection. a** Western blot analysis of Bcl-2, Bax, cleaved caspase-3, TNF-α, IL-6, pro-IL-1β, and mature IL-1β in spinal cord tissues from different groups. **b** Quantitative analysis of the protein levels shown in (**a**). Compared to the Vehicle group, intraperitoneal administration of LUT did not significantly alter the expression levels of the aforementioned apoptosis-related and inflammation-related proteins, suggesting that this treatment regimen at the tested dose failed to confer a significant anti-apoptotic or anti-inflammatory effect at the molecular level in the spinal cord. The data are presented as the mean ± *SD*. *n* = 3, ^***^*p* <0.001 vs Sham group.

**Supplementary Fig. 7 Histological analysis of spinal cord tissue by HE staining following LUT treatment via intraperitoneal injection in SCI mice.** Representative HE-stained transverse sections of the spinal cord at the lesion epicenter from different treatment groups. Compared with the Sham group, the spinal cord tissue from the Vehicle group exhibited severe structural damage, including the formation of large cavities, extensive infiltration of inflammatory cells, and loss of motor neurons. Administration of LUT via intraperitoneal injection did not markedly ameliorate these histopathological changes compared to the Vehicle group, indicating a lack of significant therapeutic effect at the tissue morphology level under this treatment regimen.

**Supplementary Fig. 8 Assessment of neuronal apoptosis in the spinal cord by TUNEL/NeuN co-staining following LUT treatment via intraperitoneal injection in SCI mice.** Representative images (**a**) and quantitative analysis (**b**) of TUNEL (green) and NeuN (red) co-staining in the spinal cord anterior horn at the lesion epicenter. Nuclei are counterstained with DAPI (blue). Compared to the Sham group, the Vehicle group showed a significant increase in the number of TUNEL-positive neurons, indicating extensive apoptosis of neurons after injury. However, the administration of LUT via intraperitoneal injection did not markedly reduce the number of TUNEL-positive neurons compared to the Vehicle group, suggesting a lack of significant anti-apoptotic efficacy on neurons under this treatment regimen. The experiment was repeated independently three times with similar results. The data are presented as the mean ± *SD*. *n* = 3, ^***^*p* <0.001.

**Supplementary Fig. 9 Assessment of microglial activation by IL-1β and Iba1 co-localization in the spinal cord of SCI mice treated with LUT.** Representative immunofluorescence images (**a**) and quantitative analysis (**b**) showing the co-localization of IL-1β (green) and Iba1 (red, a microglial marker) in the spinal cord. Nuclei are stained with DAPI (blue). Compared to the Sham group, the Vehicle SCI group exhibited a significant increase in the number of cells with IL-1β/Iba1 co-localization and the intensity of the signals. However, administration of LUT via intraperitoneal injection did not markedly reduce this co-localization compared to the Vehicle group, indicating a lack of significant suppression of microglia-mediated inflammation under this treatment regimen. The experiment was repeated independently three times with similar results. The data are presented as the mean ± *SD*. *n* = 3, ^***^*p* <0.001.

**Supplementary Fig. 10 MSN-BO@ICG exhibits brain and spinal cord targeting properties and sustained-release characteristics in uninjured mice.** Representative *in vivo* fluorescence images of uninjured mice captured at different time points (0, 12, and 24 h) after tail vein injection of ICG and MSN-BO@ICG using a small animal *in vivo* imaging system. Additionally, fluorescence accumulation in major isolated organs (heart, liver, lung, spleen, kidney, brain, and spinal cord) was examined at 24 h post-injection. Notably, the fluorescence signal of ICG was almost completely metabolized by 24 h. In contrast, MSN-BO@ICG exhibited prolonged retention and demonstrated specific targeting to the brain and spinal cord. The experiment was repeated independently three times with similar results.

**Supplementary Fig. 11 MSN-BO@ICG nanoparticles effectively cross the blood-spinal cord barrier and accumulate in the spinal cord, preferential uptake by microglia. a-c** Representative immunofluorescence images and quantitative analysis of the distribution of ICG and its colocalization with (**a**) Iba1, (**b**) GFAP and (**c**) βIII-tubulin in mice spinal cord sections following tail vein injection of different drugs. Scale bar = 50 μm. **d** Quantification of the colocalization area percentage between ICG and different cell markers. MSN-BO@ICG exhibited intense ICG signals throughout the spinal cord tissue, and the colocalization area of ICG with Iba1 was significantly higher than that with GFAP or βIII-tubulin. The experiment was repeated independently three times with similar results. The data are presented as the mean ± *SD*. *n* = 3, ^***^*p* < 0.001.

**Supplementary Fig. 12 MSN-BO@LUT treatment significantly downregulates tight junction protein expression in primary meningeal endothelial cells****.** Representative images and quantitative analysis of tight junction proteins (**a**, **b**) Claudin-5, (**c**, **d**) Occludin and (**e**, **f**) ZO-1 in primary meningeal endothelial cells of neonatal rats. Cells were treated with vehicle, LUT, MSN@LUT, or MSN-BO@LUT. The target tight junction proteins are stained in red, and nuclei are counterstained with DAPI. In the Vehicle, LUT and MSN@LUT group, all three tight junction proteins exhibited high-intensity and continuous linear distribution along the cell borders, indicating an intact and tight barrier structure. Following MSN-BO@LUT intervention, the red fluorescence signals were significantly reduced and appeared discontinuous or fragmented, suggesting a disruption of the tight junction integrity. The experiment was repeated independently three times with similar results. The data are presented as the mean ± *SD*. *n* = 3, ^***^*p* <0.001. Scale bar = 50 μm.

**Supplementary Fig. 13 The down-regulation effect of MSN-BO@LUT treatment on cerebral tight junction proteins is reversible. a** Schematic of the experimental schedule. Mice were acclimated for 7 d, followed by 10 d of intrathecal administration of MSN@LUT or MSN-BO@LUT (Drug on), and then subjected to a 7-d drug withdrawal period (Drug off). Spinal cord sections were collected at the indicated time points. **b, c** Representative images and quantitative analysis of tight junction proteins (**b**) Occludin and (**c**) ZO-1 in SCI mice. After 7-d of “Drug off”, the ZO-1 and Occludin expression level in the MSN-BO@LUT group recovered to a level comparable to that of the vehicle group. The experiment was repeated independently three times with similar results. The data are presented as the mean ± *SD*. *n* = 3, ^***^*p* <0.001. Scale bar = 50 μm.

**Supplementary Fig. 14 Cytotoxicity assessment of MSN-BO@LUT on BV2 cells.** Cell viability of BV2 cells treated with different concentrations of MSN-BO@LUT for 24 h was detected by CCK8 assay. The results indicated that LUT did not show obvious cytotoxicity at concentrations lower than 20 μg/mL, demonstrating its safety within this concentration range for subsequent cell experiments. The data are presented as the mean ± *SD*. *n* = 6, **p* < 0.05.

**Supplementary Fig. 15 MSN-BO@LUT protects co-cultured HT22 neurons from microglia-mediated apoptosis in a transwell system. a Schematic diagram of the *in vitro* transwell co-culture system. BV2 cells were seeded on the apical side and HT22 cells on the basolateral side. b Flow cytometry analysis of apoptosis in HT22 cells with different treatment conditions. c TUNEL staining (green) of HT22 cells. Nuclei are stained with DAPI (blue). d** Quantitative analysis of the TUNEL-positive cells shown in (**c**). **LPS stimulation of BV2 cells on the apical side significantly induced apoptosis in the co-cultured HT22 cells. MSN-BO@LUT significantly attenuated HT22 apoptosis, demonstrating its efficacy in inhibiting microglia-mediated neurotoxicity. The d**ata are presented as mean ± *SD*, *n* = 3. *^*^p* < 0.05, *^**^p* < 0.01, *^***^****p* < 0.001.** The experiment was repeated independently three times with similar results in (**b**, **c**).

**Supplementary Fig. 16 MSN-BO@LUT treatment ameliorates the motor behavior activity of SCI mice.** Quantitative analysis of behaviors in the open field test after 10 days of treatment. Parameters include: distance in zone, fast time in zone, mean speed in zone and resting time in zone. Mice in the Vehicle group exhibited significant anxiety-like behavior, characterized by reduced exploration of the central area. Treatment with MSN-BO@LUT significantly reversed these trends, increasing the central area exploration across all parameters, indicating a functional recovery in emotional and motor-related behaviors post-SCI. The data are presented as mean ± *SD*, *n* = 12. ^*^*p* < 0.05, ^**^*p* < 0.01, ^***^*p* < 0.001.

**Supplementary Fig. 17 *In vivo* biosafety assessment of MSN-BO@LUT in SCI mouse. a** Body weight changes of mice in different groups over the 10-day treatment period post-SCI. **b** Organ weights (heart, liver, kidney) of mice after 10 days of treatment. **c** Serum biochemical analysis after 10 days of treatment. Levels of ALT, AST, CK, UN and CREA were measured. No significant differences were observed among the groups, indicating no significant liver, kidney, or cardiac muscle toxicity. **The d**ata are presented as mean ± *SD*, *n* = 12.

**Supplementary Fig. 18 Histopathological assessment of major organs after treatment with MSN-BO@LUT.** Representative HE-stained sections of the heart, liver, and spleen from mice in different treatment groups after 10 days of therapy. No obvious pathological abnormalities, such as inflammatory cell infiltration, necrosis, degeneration, or architectural disruption, were observed in the MSN-BO@LUT treated group compared to the sham and vehicle groups. The morphology of cardiomyocytes in the heart, the hepatic lobules and portal areas in the liver, and the white and red pulp in the spleen all appeared normal. These results demonstrate that MSN-BO@LUT did not induce significant histopathological toxicity in these major organs.

**Supplementary Fig. 19 MSN-BO@LUT and MP drug safety comparison evaluation. a** Dynamic changes in body weight of mice in each group after SCI surgery. **b** Weights of the major organs (heart, liver, spleen, lung, and kidneys) of the mice in each group after 10 days of treatment. **c** Serum biochemical indicators (AST, ALT, UN, CREA, CK) of each group. **d** Histopathological assessment of major organs after treatment of each group. Scale bar = 500 μm. No significant differences were observed among the groups. **The d**ata are presented as mean ± *SD*, *n* = 12.

**Supplementary Fig. 20 Representative immunofluorescence images of the spinal cord by cleaved caspase-3/βIII-tubulin co-staining following MSN-BO@LUT treatment in SCI mice.** **Representative immunofluorescence images** **(a) and** quantitative analysis (**b**) **of the spinal cord showing cleaved caspase-3 (red, apoptotic marker) and βIII-tubulin (green, neuronal marker). Nuclei are stained with DAPI (blue). Compared to the Sham group, the Vehicle group exhibited a significant increase in the number of cleaved caspase-3/βIII-tubulin double-positive neurons. Treatment with MSN-BO@LUT markedly reduced this co-localization, demonstrating its potent anti-apoptotic effect specifically on neurons after SCI.** The experiment was repeated independently three times with similar results. The data are presented as the mean ± *SD*. *n* = 3, ^**^*p* <0.01, ^***^*p* <0.001.

**Supplementary Fig. 21 Representative immunofluorescence images of the spinal cord by IL-1β/Iba1 co-staining following MSN-BO@LUT treatment in SCI mice.** Representative immunofluorescence images (**a**) and quantitative analysis (**b**) of the spinal cord showing the co-localization of IL-1β (green) and Iba1 (red, a microglial marker). Nuclei are stained with DAPI (blue). Compared to the Sham group, the Vehicle group exhibited a significant increase in both the intensity and number of IL-1β/Iba1 double-positive cells. Treatment with MSN-BO@LUT markedly reduced this co-localization, demonstrating its potent anti-inflammatory effect by inhibiting the activation of microglia and their production of IL-1β after SCI. The experiment was repeated independently three times with similar results. The data are presented as the mean ± *SD*. *n* = 3, ^**^*p* <0.01, ^***^*p* <0.001.

**Supplementary Fig. 22 Gene Ontology enrichment analysis of DEGs from RNA-seq in primary microglia treated with LUT following LPS stimulation. Primary microglia were stimulated with LPS in the presence or absence of LUT. The Gene Ontology (GO) enrichment analysis of the resulting transcriptomic data is displayed in three categories: Biological Process (BP), Cellular Component (CC), and Molecular Function (MF). The top significantly enriched terms in each category are shown. The rich factor represents the degree of enrichment, the dot size corresponds to the number of genes (count) in each term, and the color indicates the statistical significance (-log10(pvalue)). LUT treatment significantly reversed LPS-induced alterations in key processes such as response to interleukin-1, antigen processing and presentation, and apoptotic signaling pathway, and modulated functions including cytokine receptor binding and ubiquitin-like protein transferase activity, highlighting its multi-faceted role in regulating microglial activation and immune responses.**

**Supplementary Fig. 23 Bulk RNA-seq analysis of the spinal cord in SCI mice treated with or without MSN-BO@LUT. a Volcano plot displaying the differentially expressed genes (DEGs) between the MSN-BO@LUT treated group and the Vehicle-treated SCI group. Significantly upregulated genes are marked in red, downregulated genes in blue, and non-significant genes in gray. b GO enrichment analysis of the DEGs, the top significantly enriched terms are shown. c KEGG pathway enrichment analysis of the DEGs. The most significantly enriched signaling pathways and cellular processes are displayed. The analysis reveals that MSN-BO@LUT treatment extensively modulates the transcriptome, primarily by downregulating key pathways involved in the immune and inflammatory response, antigen processing and presentation, and apoptosis, thereby promoting a restorative environment after SCI.**

c

**Supplementary Fig. 24 LUT suppresses methylation levels within the TLR4 immuno-precipitation (IP) complex in PRMT2-overexpression BV2 cells.** **a** Volcano plot of differentially methylated sites. **b** Heatmap of differential methylated peptides. Following PRMT2- overexpression in BV2 cells, samples were treated with or without LUT. TLR4 protein complexes were enriched via IP, and the eluates were subjected to mass spectrometry to profile methylation modifications. Multiple significantly downregulated sites are observed, indicating a global reduction in methylation signals within the IP products upon LUT treatment. The majority of detected methylated sites exhibit significantly decreased abundance in the LUT-treated group, suggesting that LUT effectively inhibits the PRMT2-mediated methylation process associated with the TLR4 complex.

**Supplementary Fig. 25 Schematic of the proposed mechanism by which MSN-BO@LUT alleviates SCI via targeted to PRMT2 in microglia.** Following SCI, microglia are activated to M1 polarization. This process is driven by PRMT2, which promotes TLR4 methylation, leading to the activation of the MyD88/NF-κB signaling pathway and subsequent release of pro-inflammatory cytokines (e.g., IL-1β, IL-6). This inflammatory microenvironment exacerbates neuronal apoptosis, resulting in hindlimb paralysis and neurologic impairment. After treatment, MSN-BO@LUT release LUT, directly binds to and inhibits PRMT2, thereby blocking TLR4 methylation and the downstream MyD88/NF-κB signaling cascade. This inhibition suppresses M1 polarization, reduces the release of inflammatory factors and microglial cytotoxicity, ultimately ameliorating the neuroimmune microenvironment and protecting neurons from apoptosis to promote functional recovery.

**Supplementary Tables**

**Supplementary Table 1. Mouse primer sequences in RT-qPCR**

| **Name** | **Base sequence** |
| --- | --- |
| β-actin-F | CCGCGAGTACAACCTTCTTG |
| β-actin-R | CAGTTGGTGACAATGCCGTG |
| IL-1β-F | AGCTGGAGAGTGTGGATCCC |
| IL-1β-R | CCTGTCTTGGCCGAGGACTA |
| IL-6-F | CCACTTCACAAGTCGGAGGC |
| IL-6-R | GGAGAGCATTGGAAATTGGGGT |
| TNF-α-F | CTATGGCCCAGACCCTCACA |
| TNF-α-R | TCTTGACGGCAGAGAGGAGG |
| COX2-F | CGCACAAGAAGTTGAAACCA |
| COX2-R | TACGGGGTTGTTGATTTCGT |
| iNOS-F | CTCACTGGGACAGCACAGAA |
| iNOS-R | GTGGCCTTGTGGTGAAGAGT |

**Supplementary Table 2. Specific information on the primary antibodies and dilution detected by western blot**

| **NO.** | **Products** | **Species** | **Dilution info** | **Company** | **Art.NO.** |
| --- | --- | --- | --- | --- | --- |
| 1 | IL-1β | Rabbit | 1/1000 | Abcam | ab283818 |
| 2 | Bcl-2 | Rabbit | 1/2000 | Abcam | ab196495 |
| 3 | Bax | Rabbit | 1/5000 | Abcam | ab32503 |
| 4 | IL6 | Rabbit | 1/2000 | Affinity | DF6087 |
| 5 | TNF-α | Rabbit | 1/1000 | Abcam | ab183218 |
| 6 | iNOS | Rabbit | 1/2000 | Servicebio | GB11119-100 |
| 7 | cleaved caspase-3 | Rabbit | 1/1000 | CST | 9664s |
| 8 | COX2 | Rabbit | 1/2000 | Abcam | ab15191 |
| 9 | PRMT2 | Mouse | 1/1000 | Proteintech | 66885-1-Ig |
| 10 | MyD88 | Rabbit | 1/1000 | CST | 4283s |
| 11 | Toll-like receptor 4 | Rabbit | 1/1000 | CST | 14358s |
| 12 | NF-κB | Rabbit | 1/1000 | CST | 8242s |
| 13 | phospho-NF-κB | Rabbit | 1/1000 | CST | 3033s |
| 14 | β-actin | HRP Conjugated | 1/3000 | Servicebio | ZB15001-HRP-100 |
| 15 | adme-R | Rabbit | 1/1000 | CST | 13522 |

**Supplementary Table 3. Specific information on the primary antibodies and dilution detected in immunofluorescence**

| **NO.** | **Products** | **Species** | **Dilution info** | **Company** | **Art.NO.** |
| --- | --- | --- | --- | --- | --- |
| 1 | IL-1β | Rabbit | 1/300 | Abcam | ab283818 |
| 2 | iNOS | Rabbit | 1/300 | Servicebio | GB11119-100 |
| 3 | COX2 | Rabbit | 1/300 | Abcam | ab15191 |
| 4 | Phospho-NF-κB | Rabbit | 1/300 | CST | 3033s |
| 5 | βIII-tubulin | Mouse | 1/300 | Abcam | ab78078 |
| 6 | Iba1 | Goat | 1/300 | Abcam | ab5076 |
| 7 | Neun | Mouse | 1/300 | Abcam | ab104224 |
| 8 | cleaved caspase-3 | Rabbit | 1/300 | CST | 9664s |
| 9 | NF-κB | Rabbit | 1/300 | CST | 8242s |

**Supplementary Western blot images**
